# A hybrid expert approach for retrospective assessment of occupational exposures in a population-based case-control study of cancer

**DOI:** 10.1186/s12940-019-0451-0

**Published:** 2019-02-15

**Authors:** Jean-François Sauvé, Jérôme Lavoué, Louise Nadon, Ramzan Lakhani, Mounia Senhaji Rhazi, Robert Bourbonnais, Hugues Richard, Marie-Élise Parent

**Affiliations:** 1 Department of Environmental and Occupational Health, Université de Montréal, School of Public Health, Montréal, Québec Canada; 2 Centre de recherche du CHUM, Montréal, Québec Canada; 3 INRS-Institut Armand-Frappier, Université du Québec, 531 Boul. des Prairies, Laval, Quebec H7V 1B7 Canada; 4 Department of Social and Preventive Medicine, Université de Montréal, School of Public Health, Montréal, Québec Canada

**Keywords:** Population-based studies, Occupational exposures, Retrospective exposure assessment, Expert assessment

## Abstract

**Background:**

While the expert-based occupational exposure assessment approach has been considered the reference method for retrospective population-based studies, its implementation in large study samples has become prohibitive. To facilitate its application and improve upon it we developed, in the context of a Montreal population-based study of prostate cancer (PROtEuS), a hybrid approach combining job-exposure profiles (JEPs) summarizing expert evaluations from previous studies and expert review. We aim to describe the hybrid expert method and its impacts on the exposures assigned in PROtEuS compared to those from a previous study coded using the traditional expert method.

**Methods:**

Applying the hybrid approach, experts evaluated semi-quantitative levels of confidence, concentration and frequency of exposure to 313 agents for 16,065 jobs held by 4005 subjects in PROtEuS. These assessments were compared to those from a different set of jobs coded in an earlier study of lung cancer, conducted on the same study base, for 90 blue-collar occupations and 203 agents. Endpoints evaluated included differences in the number of exposures and in the distribution of ratings across jobs, and the within-occupation variability in exposure.

**Results:**

Compared to jobs from the lung cancer study, jobs in PROtEuS had on average 0.3 more exposures. PROtEuS exposures were more often assigned definite confidence ratings, but concentration and frequency levels tended to be lower. The within-occupation variability in ratings assigned to jobs were lower in PROtEuS jobs for all metrics. This was particularly evident for concentration, although considerable variability remained with over 40% of occupation/agent cells in PROtEuS exposed at different levels. The hybrid approach reduced coding time by half, compared to the traditional expert assessment.

**Conclusions:**

The new hybrid expert approach improved on efficiency and transparency, and resulted in greater confidence in assessments, compared to the traditional expert method applied in an earlier study involving a similar set of jobs. Assigned ratings were more homogeneous with the hybrid approach, possibly reflecting clearer guidelines for coding, greater coherence between experts and/or reliance on summaries of past assessments. Nevertheless, significant within-occupation variability remained with the hybrid approach, suggesting that experts took into account job-specific factors in their assessments.

**Electronic supplementary material:**

The online version of this article (10.1186/s12940-019-0451-0) contains supplementary material, which is available to authorized users.

## Introduction

Occupational exposure assessment in community-based studies involves evaluating jobs distributed across a range of industries and workplaces, often over several decades, and with limited historical measurement data available. Self-reports, job-exposure matrices (JEMs), and expert review of jobs have traditionally been used to estimate past exposures [1]. Unlike JEMs [2–4], where the same exposure estimate is assigned to all jobs sharing the same occupational title, expert review of individual jobs descriptions uses tasks, processes and other information reported by subjects to assign job-specific exposures [5]. For this reason, the expert approach has been recognized as the reference method for retrospective community-based studies [6]. However, this labor-intensive process has become prohibitive with ever-increasing study sample sizes. Its reliability also depends on the knowledge base of the individual experts [1] and it has been criticized for the lack of transparency underlying the assessments [1, 7, 8].

Refining the expert method to increase its efficiency while maintaining its ability to provide accurate job-specific estimates of exposure represents an active area of research. This includes linking questionnaire responses with predefined decision rules to automatically classify jobs as unexposed, exposed, or necessitating further review [9–11]. Such rules can be drawn by expert judgment, but they may also be informed by existing sources of exposure information such as JEMs [12] or exposure evaluations from past studies [8, 13].

We present here an approach to retrospective exposure assessment developed for PROtEuS (Prostate Cancer & Environment Study), a population-based case-control study comprising approximately 4000 subjects in Montreal, Canada. One objective of PROtEuS is to explore potential associations between prostate cancer risk and occupational exposure to some 300 chemical and physical agents. Due to the limited resources available for reviewing over 16,000 jobs, a hybrid method was devised in which the traditional expert method was informed by historical expert evaluations from previous Montreal-based case-control studies. Towards this, the latter were summarized into profiles by occupation (“job-exposure profiles”, or JEPs). This approach was developed to not only decrease the time spent on evaluating each job, but also to enrich the industrial hygiene information readily available to experts for coding, to standardize certain coding rules, to increase inter-expert consistency, to train new experts, and to minimize the probability of experts missing exposures for complex jobs. However, since this method provides experts with exposure distributions for a given occupation, there could be a risk that their assignments would lean too close to the JEPs, thereby overlooking idiosyncrasies in specific job circumstances. We therefore conducted an evaluation of this method by comparing the exposure data underlying the backbone JEPs to those generated from the hybrid expert approach. Our a priori hypotheses were that the hybrid approach would provide higher confidence in the assessments (as more information was available to the experts), increase the number of exposures assigned to jobs (as experts would be reminded of an extensive list of potential exposures), and lead to lower variability in exposure between jobs within the same occupation (possibly resulting from clearer guidelines for coding, greater inter-rater consistency, and/or possible over-adherence to the ratings in the JEPs).

## Methods

### Development of the job-exposure profiles for PROtEuS

#### Exposure assessment source databases

The JEPs were created primarily from the exposure data of 1611 men (762 cases and 899 population controls), aged 29–75 years, that had participated in a population-based case-control study of lung cancer (referred to here as the Lung study) conducted in Montreal, Canada, in 1996–2001 [14] (Table 1). Data from an earlier Montreal-based case-control study of 19 cancer sites conducted from 1979 to 1986 [15] were added to provide complementary information on 23 agents, predominantly metals’ physical forms.

**Table 1:** Selected characteristics of the PROtEuS, Lung, and Multisite cancer studies

	PROtEuS	Multisite cancer study	Lung cancer study (male subjects)
Number of subjects^1^	4013	4263	1661
*Cases*	*1966 (49.0%)*	*3730* ^2^	*762 (45.9%)* ^*3*^
*Controls*	*2047 (51.0%)*	*533*	*899 (54.1%)*
Years conducted	2005–2012	1979–1986	1996–2001
Number of jobs	16,065	15,067	6881
*Blue-collar* ^*4*^	*9239 (57.5%)*	*11,468 (76.1%)*	*5381 (78.2%)*
*White-collar* ^*5*^	*6826 (42.5%)*	*3597 (23.9%)*	*1500 (21.8%)*
Period covered by jobs	1943–2012	1920–1986	1934–1999
Number of agents evaluated	313	293	289

1. The PROtEuS and Multisite cancer studies included only male subjects. The Lung cancer study included both male and female subjects; only figures pertaining to male subjects are reported in the table

2. Cases covered 19 different cancer sites

3. Includes 24 mesothelioma cases

4. Jobs in 4-digit CCDO unit groups classified as skilled, semiskilled, unskilled and farming occupations in the Pineo et al. socioeconomic classification [21]

5. Jobs in 4-digit CCDO unit groups classified as professionals, management, technicians, supervisors and foremen in the Pineo et al. socioeconomic classification [21]

The traditional expert-based exposure assessment method applied in these studies has been described previously [5, 15, 16]. Briefly, each study subject (or a proxy respondent) provided a lifetime occupational history during in-person interviews with trained interviewers. The occupational history covered each job held including the job title and company name, and a general occupational questionnaire containing open-ended questions collected information on tasks performed, products or equipment used, use of protective measures and descriptions of the work environment. Subjects were also asked to describe specific work circumstances that could have entailed exposure to dust, smoke fumes or gases, oils, solvents, acids, alkalis or other chemical products, and pesticides.

The collection of lifetime occupational histories also involved specialized questionnaires (*n* = 32), containing task and agent-specific questions, to help define exposures and levels for jobs in occupations with a more complex exposure profile (e.g., welders, mechanics) and lasting ≥5 years. These structured questionnaires were quite extensive, ranging from 4 to 12 pages each. A team of trained chemists-hygienists, blind to case/control status, reviewed the occupational histories to assign standardized job and industry titles for each job. Job titles were coded according to the Canadian Classification and Dictionary of Occupations (CCDO) [17] which lists nearly 8000 7-digit occupations, thereby allowing for precise job title definitions.

A team of experts then reviewed each job description to assign exposures to a predefined list of approximately 300 chemical, physical and biological agents, including mixtures (e.g. plating solutions), chemical families (e.g. polycyclic aromatic hydrocarbons, or PAHs) and general categories (e.g. pesticides). A job was considered exposed to an agent if it was present in the workplace at a level above those found in the general environment. Exposure was rated with three semi-quantitative indices: the confidence in the assessment (possible, probable, definite), the relative concentration level (low, medium, high), and its frequency, as the percentage of the working week exposed (< 5%, 5–30, > 30%). For some agents, selected occupations and circumstances representative of low, medium and high concentration served as benchmarks to standardize the exposure assessment [14, 18]. Each job was evaluated separately by two experts, blind to case-control status, and the final assessment was based on a consensus. An example of expert assessment of diesel exhaust exposure for mechanic jobs is presented in Parent et al. [15].

#### Job-exposure profiles

**Fig. 1:**
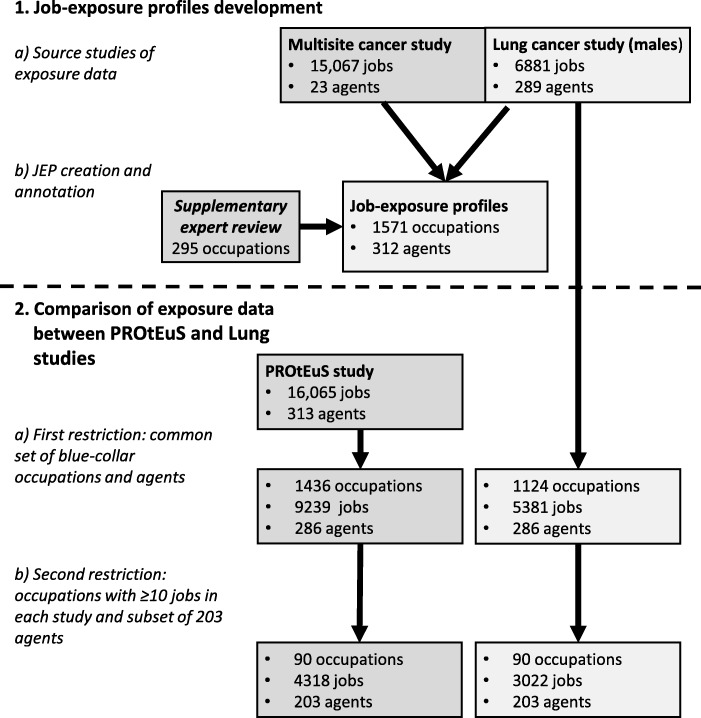
Data selection steps for JEP development and for the between-study comparisons

**Fig. 2:**
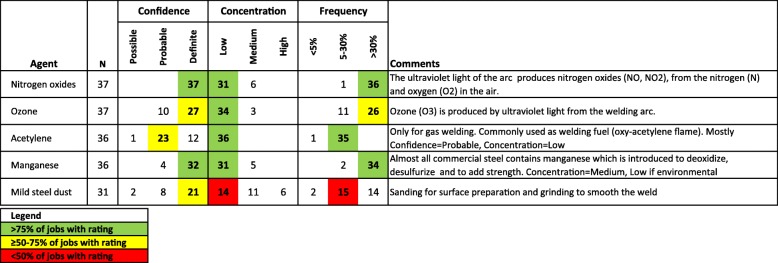
Subset of the JEP for Combination welders (CCDO 8335–126) for 5 agents

A JEP presents a comprehensive view of all exposures assigned to jobs for each occupation in the source databases. The core of the JEPs are descriptive tables summarizing the exposures assigned for 1571 7-digit occupations with at least one job evaluated in the Lung study, with exposures covering 289 agents (Fig. 1). Each summary table lists the agents in that occupation and provides the number of exposed jobs and their distribution across the confidence, concentration, and frequency categories. This distinguishes JEPs from JEMs; the latter typically assigns pre-established levels automatically while the former requires experts to select levels across categories for each exposure dimension. In JEPs, colors are used as visual clues to represent the variability in the distribution of the categorical ratings assigned to exposed jobs: green for > 75% of jobs assigned to one category, yellow for 50–75% (or based on only 2 jobs), and red for < 50% (or based on only 1 job). Additional file 1: Table S1 presents the JEP for Combination welders (i.e.*,* welders performing both gas and arc welding), with a subset of 5 agents shown in Fig. 2.

#### JEP add-ons

The JEP framework featured the following additional components to further assist the experts:

##### Job title definitions

To facilitate the linkage of a JEP to a job description, the interface enabled to search through the 8000 unique CCDO occupation codes, and to compare their descriptions (typically about 100 words). This also served as a reminder of usual activities within occupations, providing additional cues for coding.

##### Agent definitions

A brief definition of each agent was provided, including its physicochemical properties, sources and uses, correlated exposures, and relevant historical measurements from the literature, when available.

##### Annotations

The JEPs also summarized the comments or justifications made by the experts when assigning specific exposures in previous studies. A comprehensive review was conducted in PROtEuS for 295 occupations with a complex exposure profile to add comments based on prior knowledge or updated industrial hygiene literature. These guidelines provided further assistance to experts for adjusting exposure levels to specific tasks and circumstances reported by subjects, thereby improving on the transparency of assessments. For example, auto mechanics would generally be exposed to asbestos at low concentrations, but an annotation indicated that a job entailing brakes repair should be assigned medium concentration.

##### Link to source data

The experts could access the original exposure assessments of individual jobs from the source studies to compare occupational circumstances.

### Application of the hybrid expert approach in a case-control study of prostate cancer

#### PROtEuS population

The PROtEuS population has been described in detail previously [19, 20]. Briefly, eligible subjects were men aged ≤ 75 years at diagnosis or recruitment, Canadian citizens and residing in the greater Montreal area. Eligible cases were all patients newly diagnosed with prostate cancer from September 2005 to December 2009 across the main Montreal hospitals serving the French-speaking population. Controls were randomly selected from the electoral list of French-speaking electors, frequency-matched to cases on age (±5 years). Successful interviews were conducted with 1966 cases and 2047 controls. Proxy respondents (< 4%) completed the interview when subjects were unable to do so.

#### Data collection

In-person interviews elicited detailed information on socio-demographic and lifestyle factors, and a lifetime occupational history. The latter was collected using the same approach as described earlier for the source studies, including a general occupational questionnaire for each job, supplemented by specialized questionnaires, except that detailed job descriptions were obtained only for jobs lasting two years or more.

#### Application of the hybrid expert approach

The development of the JEP interface took approximately one year of part-time work by an industrial hygienist (LN) while the recruiting of subjects was ongoing. Four experts then carried out the exposure assessment in PROtEuS over two years. Of these, two were senior experts who participated in the development of the traditional expert method in the 1970s and had also applied it in the two source studies.

Exposure assessment methods in PROtEuS were analogous to those of the source studies with two exceptions. First, the frequency category for > 30% of the workweek exposed was split in two in PROtEuS: one for > 30 to 90% of the workweek, and another for > 90% to reflect continuous exposure. The second difference with earlier studies was the availability of JEPs to guide the experts’ evaluations.

When evaluating exposures for a job, experts could retrieve the JEP relevant to the 7-digit occupation title and assign exposures using both the job description and the information in the JEP. In contrast to a JEM, which assigns a fixed set of exposures and levels based on the occupation, the experts could change exposure levels for all dimensions suggested by the JEP. They could also omit exposures or assign additional ones not listed in the JEP, and combine information from several occupations when relevant to a job description. For example, a janitor reporting regular plumbing tasks could entail the use of JEPs from plumbing occupations to cover a wider spectrum of exposures. The experts could also duplicate the exposures between jobs to speed up the coding, for instance when assessing separate jobs in identical work environments for the same subject. When evaluating a job with no JEP available, the experts could use data from related occupations for guidance.

### Comparison of the exposures assigned in PROtEuS to those from a source study coded using the traditional expert method

We examined the number of exposures assigned to jobs, the confidence rating and the variability in exposure ratings between jobs within an occupation. The exposures assigned in PROtEuS were compared to those in the Lung study serving as a reference. Some comparisons were based on individual jobs, others on jobs summarized by a combination of occupation and agent (or “cells”).

Since the exposure data from the Lung study constituted a significant source of information for the experts in PROtEuS, and since the two studies represent a different sample of subjects and job histories, this comparison does not aim to examine the reliability of the hybrid method. This would have required comparing exposures assigned independently with both methods over a common sample of job descriptions. Nor can these comparisons be used to infer validity, which would have required comparisons with hygiene measurements. However, these comparisons can provide insights on macro-level differences (i.e., by broad occupation group or agent) in exposure codings between the two approaches.

#### Data selection

We restricted the comparisons to blue-collar occupations since white-collar occupations were generally exposed to fewer agents. Blue-collar occupations were those classified as skilled, semiskilled, unskilled and farming occupations in the Pineo-Porter-McRoberts socioeconomic classification [21, 22], which represented 1124 of all JEPs (Fig. 1). We then restricted the data to the 90 occupations that had at least 10 jobs evaluated in both studies (listed in Additional file 1: Table S2), representing 4318 jobs in PROtEuS and 3022 in the Lung study. To avoid including agents with a very small number of exposed jobs, the comparisons were based on 203 agents (listed in Additional file 1: Table S3) that were evaluated in both studies and that had ≥ 5% of jobs exposed in at least one of the 90 occupations in any one of the two studies.

#### Comparisons based on individual jobs and exposures

We first computed the average and selected quantiles for the number of exposures by job in PROtEuS and in the Lung study. We then compared the relative distribution of the confidence ratings of individual exposures coded (e.g., each of the 58 combination welder jobs exposed to nitrogen oxides in Fig. 2). Since a job could be exposed to several agents, the number of data points in the analysis was greater than the total number of jobs. We also applied a cumulative logistic model on the ordinal confidence ratings of exposed jobs, using the study as a covariate with the Lung study representing the reference level and PROtEuS representing the other level. This model provided a quantitative measure of the differences in the relative distribution of confidence ratings assigned between the studies, expressed as a cumulative odds ratio (OR). A cumulative OR of 2 can be interpreted as a two-fold increase in the odds of an exposed job coded with probable or definite confidence (relative to possible), or with definite confidence (relative to possible or probable) in PROtEuS compared to the Lung study. We also applied this model to the concentration and frequency indices. We combined the frequency categories for 30–90% and > 90% of the workweek exposed in PROtEuS to match the format used in the Lung study.

#### Comparisons based on cells

To compare the proportion of jobs exposed (i.e., prevalence) to an agent in an occupation between the hybrid approach and the traditional expert method, we summarized the exposure data by combination of occupation and agent for both studies separately. We assessed if cells with non-null exposure in PROtEuS (defined as ≥5% of jobs exposed) also had non-null exposure in the Lung study, representing concordance in exposure status. We also evaluated the prevalence among concordant exposed cells in PROtEuS relative to the Lung study, and the correlation in the prevalence of cells using Kendall’s rank correlation coefficient. We assessed if the trends remained with other thresholds defining non-null exposure (> 0%, ≥ 10%, ≥ 25% or ≥ 50%).

To assess if the experts using the hybrid approach tended to decrease the within-occupation variability in the categorical ratings of jobs by selecting levels with the highest prevalence in JEPs (flagged in green), we categorized the relative percentage of jobs assigned to one rating with the following scheme: < 50%, 50–75%, > 75- < 100 and 100%. The first three categories matched the color codes showing the variability in exposure in JEPs, with an additional fourth category representing complete homogeneity. We compared the distribution of cells by category of relative percentage of jobs assigned to one rating in PROtEuS and in the Lung study among concordant-exposed cells. Since the proportions for cells with few jobs can only take a narrow range of values (e.g., 50% or 100% in a single rating if based on 2 exposed jobs), the comparison was restricted to cells with at least 5 exposed jobs in each study.

#### Subanalyses

We also evaluated if the trends observed in the distribution of probability and of categorical ratings of jobs remained after changing the following parameters: (1) Restricted to occupations with an expert-annotated JEP; (2) Stratified by chemical/physical group; (3) Stratified by 2-digit CCDO major group; (4) Stratified by employment period, either restricted to the years with at least 500 blue-collar jobs in both studies (1953–1993), or between two periods split at the midpoint of the years covered by the job histories (1934–1972 and 1973–2012).

## Results

In PROtEuS, the experts assessed a total of 16,065 jobs held by 4005 subjects. The average age of subjects at interview was 65 years (interquartile interval 61–70 years). On average, the experts evaluated 4 jobs per subject (range 1–13 jobs) and took one hour per job to review and assign exposures. The job histories covered 2263 7-digit occupations, of which 1122 (50%) had a JEP available. The remaining 1141 occupations (50%) without a specific JEP represented 19% of all jobs (*n* = 3047). For 2385 of those jobs (78%), the experts used exposure information from at least one JEP from a different occupation, generally within the same 4-digit unit group.

Using the hybrid approach, 313 agents had at least one job exposed. 12,162 jobs (76% of total) were exposed to at least one agent. Blue-collar jobs were more likely to be exposed to at least one agent than white-collar jobs (89% vs. 58%) and had on average 10.9 agents with some exposure (4.2 for white collar). Table S3 in Additional file 1 presents the proportion of jobs exposed to each of the 313 agents, overall and stratified by blue/white-collar status. The most prevalent agents were volatile organic liquids (39% of jobs exposed), C5-C17 alkanes (27%), organic solvents (23%), and any PAH (19%). Only 28 agents had a higher prevalence in white-collar jobs, including inks (+ 1.8%), calcium sulfate (+ 2.3%), and calcium carbonate (+ 5.7%), the latter primarily found in teaching occupations from the use of chalk. The experts assigned exposure with definite confidence 59% of the time, compared to 28% for probable and 13% for possible; the proportion of exposures with definite confidence was higher among blue-collar jobs (60%) compared to white-collar (55%).

### Comparison of the exposures assigned in PROtEuS to those from a source study coded using the traditional expert method

Comparing the exposures assigned to jobs in the two studies, one endpoint evaluated was if the use of the hybrid approach translated into more agents with exposures assigned to jobs. Among the 203 agents included in the comparisons, the average number of exposures per job was 7.7 in PROtEuS (median 5, interquartile interval 2–11), and 7.4 in the Lung study (median 5, interquartile interval 3–9). Figures were slightly higher in the 75 occupations with an expert-annotated JEP (average 9.0 agents/job in PROtEuS, 8.5 in the Lung study). The difference was greater for jobs in the period 1934–1972 (average PROtEuS 8.0, Lung study 7.2) compared to 1973–2012 (PROtEuS 7.6, Lung 7.7).

The proportion of all jobs (*n* = 4318 for PROtEuS, *n* = 3022 for Lung study) exposed to each of the 203 agents retained is presented in Additional file 1: Table S3. The rankings of agents by their prevalence of exposure were highly correlated between the two studies (Kendall correlation coefficient of 0.81). Diesel engine emissions was the most prevalent agent in PROtEuS and ranked second in the Lung study, both with 31%. Leaded engine emissions had the highest prevalence among Lung study jobs with 42%, and ranked third for PROtEuS jobs with 29%.

**Fig. 3:**
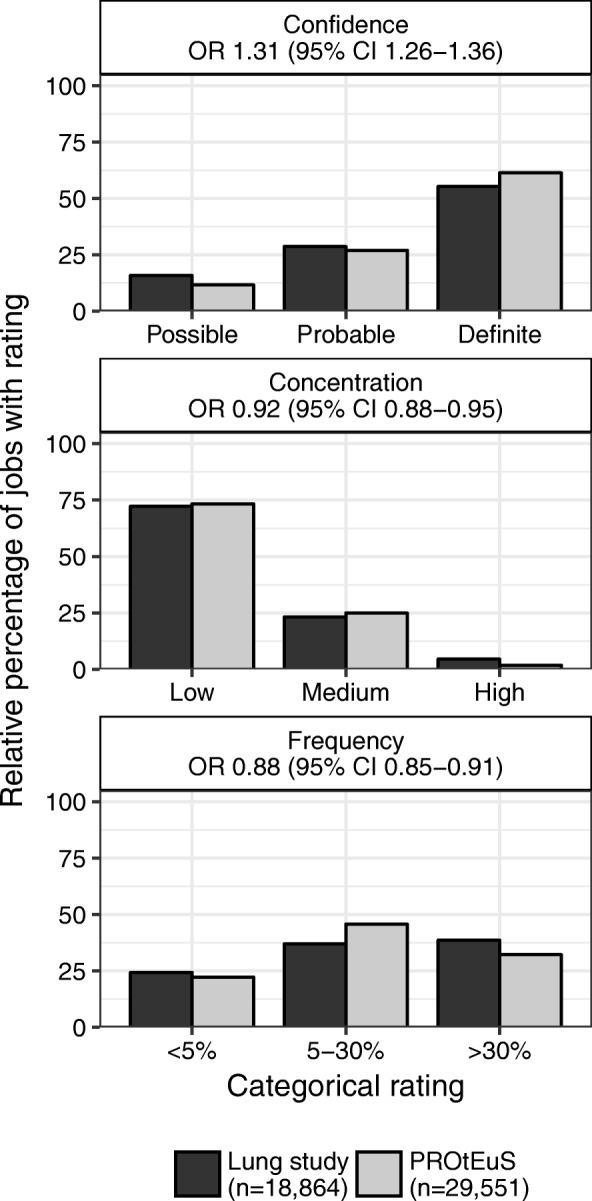
Relative distributions of the exposures assigned to jobs by categorical exposure metric between studies. OR: Cumulative odds ratio for the odds of a job exposed at higher level categories (eg., definite confidence) relative to lower level categories (eg., possible or probable confidence). CI: Confidence interval

To evaluate whether experts tended to assign exposure with higher confidence in PROtEuS, we compared the relative distribution of the confidence categories of the exposed job/agent pairs (*n* = 29,551) with those from the Lung study (*n* = 18,864) (Fig. 3). The proportion of exposures with definite confidence was higher among PROtEuS jobs (61%) compared to the Lung study (55%), with fewer ratings of possible (12% vs. 16%) and probable (27% vs. 29%). The associated cumulative OR for the odds of jobs being assigned higher reliability ratings in PROtEuS relative to the Lung study was 1.31 (95% CI 1.26–1.36). Analyses stratified by agent group, CCDO major group, and employment period (see Additional file 1: Table S4) showed comparable trends except for Sales Occupations with lower confidence in PROtEuS (OR 0.81, 95% CI 0.68–0.96). On other hand, PROtEuS jobs tended to have more jobs assigned to lower concentration (OR 0.92, 95% CI 0.88–0.95) and frequency (OR 0.88, 95% CI 0.85–0.91) ratings.

We found a high level of agreement in the binary exposure status of cells (n = 18,270): 86% of cells had a prevalence < 5% in both PROtEuS and Lung (concordant unexposed pairs), and 8% had a prevalence ≥ 5% in both studies (concordant exposed pairs) (Table 2). Discordance was twice likely to reflect exposure in the Lung study and none in PROtEuS (4% vs. 2%), although the percentages were closer with other thresholds on the minimum prevalence for non-null exposure. Among concordant exposed pairs (*n* = 1502), PROtEuS cells had a median increase of 1% in exposure prevalence relative to the Lung study (interquartile interval − 8 to + 13%). This trend of higher prevalence in PROtEuS was less sensitive to the choice of threshold except for *P* > 0% (median difference of − 0.2%). Patterns in the distribution of concordance/discordance in exposure status and in the prevalence of exposure of cells were generally similar in stratified analyses (presented in Additional file 1: Table S5).

**Table 2:** Agreement in exposure status among cells (*n* = 18,270) by threshold for minimum prevalence defining exposure

	Percent concordant		Percent discordant		Concordant exposed occupation-agent combinations		
Prevalence threshold (1)	Exposed (%)	Unexposed (%)	Exposed Lung (%) (2)	Exposed PROtEuS (%) (3)	Number of combinations (4)	Kendall correlation in probability (5)	Median difference. in probability (%), PROtEuS-Lung (6)
*P* > 0%	11.8	76.8	6.0	5.4	2162	0.61	−0.2
*P* ≥ 5%	8.2	85.5	4.1	2.2	1502	0.55	1.3
*P* ≥ 10%	6.2	90.2	2.2	1.4	1127	0.52	2.4
*P* ≥ 25%	3.7	94.2	0.9	1.1	685	0.43	2.9
*P* ≥ 50%	2.2	96.3	0.6	0.9	403	0.36	2.3

1. Minimum prevalence (proportion of jobs exposed) in cell to be considered exposed

2. Proportion of cells with non-null exposure in Lung and null exposure in PROtEuS

3. Proportion of cells with non-null exposure in PROtEuS and null exposure in Lung

4. Number of concordant exposed occupation-agent combinations

5. Kendall correlation in the probability of exposure between concordant exposed cells

6. Median difference in probability (probability in PROtEuS minus probability in Lung) across concordant exposed cell

**Fig. 4:**
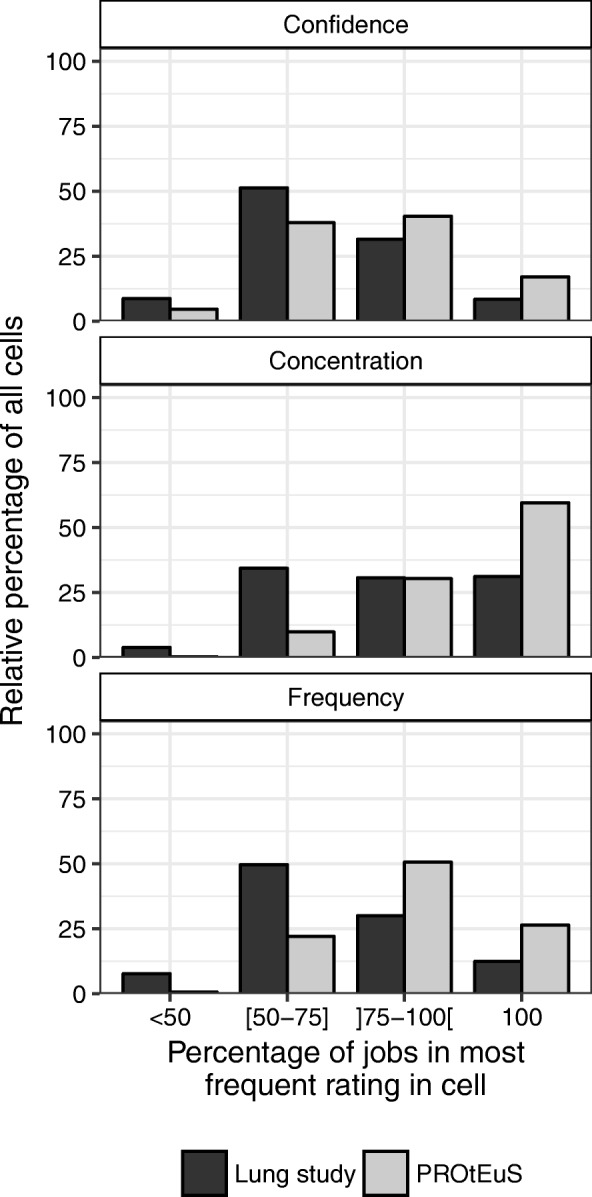
Relative proportions of jobs in the most frequently-assigned rating across concordant exposed cells by study and metric

We also evaluated the influence of the exposure assessment method on the variability in the categorical ratings of exposed jobs within a cell. There was a clear trend of more jobs assigned to the same rating in PROtEuS compared to exposures coded using the traditional method for all metrics (Fig. 4). This effect was greater for concentration where 60% of cells in PROtEuS had all jobs assigned the same level, compared to 31% in the Lung study.

## Discussion

The hybrid expert approach built on existing exposure evaluations as a tool to shorten the expert-based retrospective exposure assessment for a large scope of agents in a community-based study. It benefits from the positive attributes of a JEM by streamlining homogeneous exposure profiles while allowing the experts to account for the idiosyncrasies of specific jobs. Since the hybrid approach was based on the exposure data of individual jobs, JEPs could be developed for very precise occupational groups, featuring an extensive set of agents and containing expert annotations. The use of a JEM as a set of initial exposure assessments that can be adjusted by experts according to job descriptions had been reported previously in the literature [23]. However, unlike the current approach, experts used pre-selected exposure levels from the JEM, rather than exposure distributions.

Other recent strategies that combines individual job-by-job assessment and the group-based assignment of JEMs in population studies include predefined decision rules [10] and evaluations restricted to a sub-sample of all jobs [24]. Among those, the hybrid approach leans closer to the traditional expert method as it involves the review of each job description, although guided by past data. Its use of open-ended questions and narrative job descriptions (except for specialized questionnaires) also makes it less suitable to the application of rule-based approaches to assign exposure estimates. The development of text-mining methods could help to link narrative job descriptions to programmable decision rules [25]. However, as with the development of decision rules, the keywords and scenarios to extract from narrative job descriptions need to be defined a priori and may miss some less common or unusual exposure circumstances [25] that could have been identified using expert review. Moreover, the use of open-ended questions can in theory allow for post hoc assessment of additional exposures beyond those initially evaluated [16], compared to the use of direct, agent-specific questions.

The feedback of PROtEuS experts using this approach has been positive, from both senior experts who had a long history of applying the traditional approach, and those who were new to exposure assessment in population-based studies. It provided experts with more structure, guidance, and readily accessible information, while allowing them complete latitude in their assignments. The JEPs also represented a useful tool for training junior experts. While time was invested in developing this approach and its interface, it resulted in an estimated two-fold reduction in the experts’ time to carry out their assignment of job/industry titles and exposures. One source of time saving relates to the color schemes used in the JEPs, whereas the green colorings associated with highly homogeneous assignments in earlier studies allowed experts to focus on more heterogeneous exposures between jobs. The larger proportion of white-collar jobs in PROtEuS compared to earlier studies may have also contributed to the shorter overall coding time as they tended to be associated with fewer exposures.

The experts involved in assessing exposures in the Lung study also had access to past data in the form of crude summaries of the exposure data from the Multisite study by occupation (with less precise 4-digit CCDO codes). They also occasionally used a small sample of jobs within an occupation for guidance. However, these exposure sources were inconsistently used for lack of a comprehensive computerized interface. In contrast, the hybrid approach represents a systematic application of available and enhanced exposure information in a study.

The comparison of the exposures assigned with the hybrid approach to those coded with the traditional expert method was not designed to appraise its reliability or its validity. This would have required a comparison based on independent assessments of the same jobs using both approaches, [26, 27] or to job descriptions for which industrial measurements are available [28]. Nevertheless, our group has also applied a simplified version of the hybrid approach to evaluate occupational exposure to engine emissions in other Canadian population studies, where expected exposure-cancer associations were observed [29, 30].

When comparing exposures assigned to jobs in selected blue-collar occupations, the average number of agents with exposure by job was slightly higher in PROtEuS. The contrast was clearer for confidence where the experts assigned exposures as definite more frequently with the hybrid approach, probably owing to the wealth of information available in JEPs including the annotated guidelines. Regarding the concentration and frequency of exposure, PROtEuS jobs were more likely to be assigned to lower categories (e.g., low concentration) although this pattern was less consistent in analyses stratified by chemical group compared to confidence. Lower concentrations levels might reflect improved work conditions in the PROtEuS study era, as compared to the Lung’s. The comparatively high variation for frequency of exposure is in line with the observations of Friesen et al, [13, 31] where frequency was the metric with the lowest agreement between ratings assigned to jobs by decision rules and those assigned by experts.

The high concordance in exposure status in analyses stratified by cell suggests that when there was some exposure in JEP, experts were also likely to assign exposure in PROtEuS, and analogously for no exposure. For the discordance in exposure status, no clear trend emerged since the difference between the proportion of cells only exposed in the Lung study or in PROtEuS varied with the threshold used on minimum prevalence defining exposure.

As anticipated, we observed decreased within-occupation variability in exposure for jobs coded with the hybrid approach, especially for the index of concentration where nearly 60% of cells had all jobs exposed at the same level in PROtEuS (31% in the Lung cancer study). The tendency towards greater homogeneity can be interpreted in two contrasting ways. On one hand, this may represent clearer guidelines for coding and higher coherence in the ratings of jobs for similar exposure scenarios. On the other hand, this may also result from the experts putting a higher weight on past data in their judgment compared to the specificities of the individual job descriptions. However, findings of higher confidence, but lower frequency or concentration ratings, argue against a tendency for systematic compliance to the JEPs. Nevertheless, there remained significant variability in the ratings assigned for most cells, suggesting that the experts integrated both sources of information in their assessments, as shown by the between-study differences in the distribution of categorical ratings of jobs.

While the comparisons aimed to evaluate differences in exposures assigned using two related methods, they were performed on jobs from two different study populations, distributed in different occupations, and held at somewhat different times which may confound the trends observed. However, both studies were conducted in the Montreal region, and some of these differences were mediated by restricting the comparisons to a set of common occupations. Moreover, sensitivity analyses stratified by employment period or by occupation group, where residual differences in study populations should be further reduced, yielded comparable results.

The comparisons of exposures assigned to jobs using the two approaches were also limited to fewer than 10% of all blue-collar 7-digit CCDO codes of jobs in our study population. These occupations were however highly prevalent, representing approximately half of all blue-collar jobs evaluated. Some of the trends observed may also result from residual differences in the distribution of jobs across occupations between the studies, even after restricting the comparisons to the most prevalent blue-collar occupations.

One limitation of the hybrid approach applied here is the lack of systematic information on time trends in exposure, which was provided only in the comments and in the definition of benchmark concentration levels for some agents. Potential improvements for future developments of the approach could include providing period-specific JEPs. Another limitation is the use of categorical ratings for concentration, reflecting the information available in the source databases. Several studies have developed methods to derive quantitative estimates of retrospective exposures in population studies, [32, 33] including in Montreal [34]. In practice, however, very few agents would have a large number of relevant historical measurements to support the development of quantitative estimates, limiting the ability to got beyond categorical ratings.

The development of the hybrid approach was contingent of the availability of a large pool of exposure data spanning a wide range of occupations, and of specialized expertise to interpret and augment this information with comments and guidelines. Our team could source data from two large case-control studies conducted in the same region, and two experts had over 20 years of experience in implementing the traditional expert method. However, significant data gaps remained since half of all 7-digit occupations encompassed by PROtEuS jobs had no specific JEP available. The additional exposure data collected for these less prevalent occupations could improve the coverage of the population in future studies using the hybrid approach. For the availability of data to create profiles, the CANJEM matrix (available from www.canjem.ca) [35, 36] summarizing expert evaluations from studies conducted in Montreal (including the two source studies of JEPs) may constitute an initial source of information to implement the hybrid approach for other investigators.

Our evaluation of the hybrid expert approach suggests that it retains the desirable qualities of expert review by tailoring the assessment to individual job descriptions and with greater confidence. It thereby circumvents the main limitation of JEMs. However, as it relies on experts, it may not be applicable in the context of very large investigations, where a compromise towards JEMs is often the only feasible option. The new approach should be of interest for future community-based studies interested in assessing efficiently a wide range of agents, which other recent methods based on automated decision rules cannot easily do. If judged necessary, adjustments could be made to the job exposure profiles to reflect country-specific occupational exposure circumstances.

## Conclusion

In summary, the application of the hybrid expert approach decreased the time required to evaluate exposures and increased the confidence of exposures assigned to jobs compared to jobs coded with the traditional expert method. It also reduced the variability in the ratings of jobs exposed to an agent within the same occupation, although whether this reflects greater coding coherence, over-influence of JEPs, or both, is unclear. Nevertheless, as there remained considerable variability in exposure ratings within jobs, the method overcomes the major limitation of JEMs by allowing to take into consideration job specificities. Finally, a valuable advantage of the hybrid approach is the greater transparency in the assessment represented by the exposure information assembled and of the overall coding rules used by the experts to assign exposures, which can help with the interpretation of findings using this approach.

## Additional file

Additional file 1: **Table S1.** Example of job-exposure profile for combination welders (CCDO 8335–126). **Table S2.** List of the 90 blue-collar occupations included in the analysis. **Table S3.** Proportion of jobs exposed by agent in PROtEuS (*n* = 313), overall and stratified by blue/white-collar status, and proportion of PROtEuS and Lung cancer study jobs exposed among the blue-collar occupations (*n* = 90) and agents (*n* = 203) retained in the comparison. **Table S4.** Cumulative odds ratios and 95% confidence intervals for the association between the categorical confidence, concentration or frequency of exposed jobs and source of exposure data. **Table S5.** Agreement in exposure status among cells between Lung and PROtEuS exposure data. (DOCX 144 kb)

## Abbreviations

CCDO: Canadian Classification and Dictionary of Occupations
CI: Confidence interval
JEM: Job-exposure matrix
JEP: Job-exposure profile
OR: Odds ratio
PAH: Polycyclic aromatic hydrocarbon(s)
