# Supplementary material for: A hybrid expert approach for retrospective assessment of occupational exposures in a population-based case-control study of cancer
Source: Environ Health. 2019 Feb 15;18:14. doi: 10.1186/s12940-019-0451-0 (PMC6377721; doi:10.1186/s12940-019-0451-0)
Supplement: Supplementary file 1 — Table S1. Example of job-exposure profile for combination welders (CCDO 8335–126). Table S2. List of the 90 blue-collar occupations included in the analysis. Table S3. Proportion of jobs exposed by agent in PROtEuS (n = 313), overall and stratified by blue/white-collar status, and proportion of PROtEuS and Lung cancer study jobs exposed among the blue-collar occupations (n = 90) and agents (n = 203) retained in the comparison. Table S4. Cumulative odds ratios and 95% confidence intervals for the association between the categorical confidence, concentration or frequency of exposed jobs and source of exposure data. Table S5. Agreement in exposure status among cells between Lung and PROtEuS exposure data. (DOCX 144 kb) [file 12940_2019_451_MOESM1_ESM.docx]

**A hybrid expert approach for retrospective assessment of occupational exposures in a population-based case-control study of cancer**

**Jean-François Sauvé ^1,2,3^, Jérôme Lavoué ^1,2^, Louise Nadon ^3^, Ramzan Lakhani ^3^, Mounia Senhaji Rhazi ^3^, Robert Bourbonnais ^3^, Hugues Richard^,3^, Marie-Élise Parent ^2,3,4^**

1. Université de Montréal, School of Public Health, Department of Environmental and Occupational Health, Montréal, Québec, Canada
2. Centre de recherche du CHUM, Montréal, Québec, Canada
3. INRS-Institut Armand-Frappier, Université du Québec, Laval, Québec, Canada
4. Université de Montréal, School of Public Health, Department of Social and Preventive Medicine, Montréal, Québec, Canada

Additional file 1

**Table S1.** Example of job-exposure profile for combination welders (CCDO 8335-126)

p.2

**Table S2**. List of the 90 blue-collar occupations included in the analysis

p.7

**Table S3.** Proportion of jobs exposed by agent in PROtEuS (n=313), overall and stratified by blue/white-collar status, and proportion of PROtEuS and Lung cancer study jobs exposed among the blue-collar occupations (n=90) and agents (n=203) retained in the comparison

p.10

**Table S4.** Cumulative odds ratios and 95% confidence intervals for the association between the categorical confidence, concentration or frequency of exposed and source of exposure data

p.19

**Table S5.** Agreement in exposure status among cells between Lung and PROtEuS exposure data

p.21

Table S1. Example of job-exposure profile for combination welders (CCDO 8335-126)

**Occupation title description:** Welds metal parts together to fabricate or repair parts and equipment, using gas and arc welding equipment: Cleans grease and corrosion from workpiece, using solvent, wire brush and portable grinder. Examines workpiece for defects. Chips or grinds away metal around cracks and joints in workpiece to enable complete fusing of weld. Heats and straightens bent pieces, using torch, hammer, tongs and anvil. Positions and clamps parts together in vise, jig or holding fixture preparatory to welding. Connects hoses to gas supply and selects torch tip or inserts electrode into portable holder and clamps cable to workpiece. Sets pressure gauges on gas supply lines or sets current and voltage levels on power supply. Opens gas valves on torch, ignites torch, and adjusts oxygen and fuel gas mixture to provide proper flame or strikes arc and guides torch or electrode along weld line at proper rate and angle. Builds up parts by welding layers onto worn or damaged areas. Removes slag splatter and excess weld, using chipping hammer, wire brush and portable grinder. Welds in flat, vertical or overhead welding positions. Cuts metal shapes, using flame-cutting torch. Gives directions to other workers helping with welding activities. May layout, fit and align metal parts, using chalk, scriber. center punch and square.

**Legend**: Green: >75% of exposed jobs with rating; Yellow: 50-75% of exposed jobs with rating; Red: <50% of exposed jobs with rating,

**Number of jobs assessed in prior studies**: Multisite cancer study: **61**; Lung cancer study: **37**

| **Agent** (* denotes agent with exposure data from Multisite study) | **N exposed jobs** | **Confidence** | | | **Concentration** | | | **Frequency** | | | **Comments** |
| --- | --- | --- | --- | --- | --- | --- | --- | --- | --- | --- | --- |
|  |  | **Possible** | **Probable** | **Definite** | **Low** | **Medium** | **High** | **<5%** | **5-30%** | **>30%** |  |
| *Iron fumes | 58 |  | 1 | 57 | 2 | 20 | 36 | 1 | 10 | 47 | This is the most likely base metal in construction steel AND stainless steel. The concentration is high in most cases |
| *Manganese fumes | 57 |  | 1 | 56 | 21 | 36 |  | 2 | 10 | 45 | Almost all commercial steel contains manganese which is introduced to deoxidize, desulfurize and to add strength. Concentration=medium, low if environmental |
| Nitrogen oxides | 37 |  |  | 37 | 31 | 6 |  |  | 1 | 36 | The ultraviolet light of the arc produces nitrogen oxides (NO, NO2), from the nitrogen (N) and oxygen (O2) in the air. |
| Ozone | 37 |  | 10 | 27 | 34 | 3 |  |  | 11 | 26 | Ozone (O3) is produced by ultraviolet light from the welding arc. |
| Arc welding fumes | 37 |  | 9 | 28 |  | 26 | 11 |  | 2 | 35 | During arc welding |
| Iron | 37 |  | 2 | 35 | 22 | 15 |  |  | 2 | 35 | This is the most likely base metal in construction steel AND stainless steel. The concentration is high in most cases |
| Ultraviolet radiation | 37 |  | 10 | 27 | 37 |  |  |  | 11 | 26 | Arc welding. Higher with Aluminum and Stainless steel welding |
| Carbon monoxide | 36 |  |  | 36 | 30 | 6 |  |  | 1 | 35 | Due to "breakdown" of atmospheric CO2 and formed by the incomplete combustion of the electrode covering or flux and by the use of carbon dioxide CO2 as a shielding gas; low concentration |
| Acetylene | 36 | 1 | 23 | 12 | 36 |  |  | 1 | 35 |  | Only for gas welding. Commonly used as welding fuel (oxy-acetylene flame) Mostly probable confidence, low concentration |
| Gas welding fumes | 36 | 1 | 9 | 26 |  | 28 | 8 | 1 | 3 | 32 | When welding with gas |
| Metal oxide fumes | 36 |  |  | 36 | 6 | 19 | 11 |  |  | 36 | Oxidized metal during welding. Individual metal (Fe, Cr, Ni, etc.) oxide depends on the work piece. |
| Manganese | 36 |  | 4 | 32 | 31 | 5 |  |  | 2 | 34 | Almost all commercial steel contains manganese which is introduced to deoxidize, desulfurize and to add strength. medium concentration, low if environmental |
| Titanium | 35 | 34 |  | 1 | 35 |  |  | 1 | 4 | 30 | From TiO2 fumes (Rutile flux 6011, 6013, 7014, 7024) |
| Magnetic fields | 35 |  | 9 | 26 | 10 | 13 | 12 |  | 8 | 27 | Arc welding |
| Hydrogen fluoride | 34 | 26 | 7 | 1 | 31 | 3 |  |  | 3 | 31 | Exposure from coated flux. Common electrodes for this exposure are E7016, 7018, 7028 |
| Metallic dust | 33 | 2 | 11 | 20 | 16 | 9 | 8 | 1 | 18 | 14 | Grinding to smooth the weld or at the time of finishing |
| Abrasives dust | 32 | 2 | 14 | 16 | 4 | 12 | 16 | 3 | 29 |  | Grinding to smooth the weld finishing This includes aggregate material containing Alumina (most likely) Silicon Carbide or silica with binders like vitrified glass, resins or rubber. |
| Alumina | 32 | 4 | 22 | 6 | 15 | 17 |  | 3 | 28 | 1 | Very common abrasive grit for grinding to prepare the surface and to smooth the weld |
| Calcium oxide fumes | 32 | 25 | 7 |  | 29 | 2 | 1 |  | 4 | 28 | Basic flux electrode like H7018 or 7011 |
| Mild steel dust | 31 | 2 | 8 | 21 | 14 | 11 | 6 | 2 | 15 | 14 | Sanding for surface preparation and grinding to smooth the weld |
| Chrysotile asbestos | 30 | 19 | 10 | 1 | 28 | 2 |  | 1 | 8 | 21 | Slag from electrodes and gloves |
| *Nickel fumes | 30 |  | 6 | 24 | 4 | 26 |  | 11 | 16 | 3 | Part of welding fumes, if base metal is stainless steel or high alloy steel with Nickel |
| Mineral-based oil & grease pyrolysis fumes | 29 | 3 | 15 | 11 | 9 | 19 | 1 | 1 | 9 | 19 | Presence of oil on mild steel to prevent rusting. Pyrolyzed during welding. Common in construction industries. |
| Silicon carbide | 28 | 16 | 7 | 5 | 13 | 15 |  | 3 | 25 |  | Very common abrasive grit for grinding to smooth the weld |
| *Chromium fumes | 28 |  | 5 | 23 | 3 | 24 | 1 | 10 | 15 | 3 | Part of welding fumes, if base metal is stainless steel or high alloy steel with Chromium |
| Organic solvents | 26 | 4 | 6 | 16 | 10 | 15 | 1 | 1 | 20 | 5 | Metal degreasing before welding |
| Pulsed electromagnetic fields | 26 | 1 | 9 | 16 |  | 26 |  | 1 | 19 | 6 | Use of grinder and arc welding |
| Fluorides | 23 | 21 | 1 | 1 | 22 | 1 |  |  | 3 | 20 | Exposure from coated flux. Common electrodes for this exposure are E7016, 7018, 7028 |
| Chromium | 23 | 4 | 5 | 14 | 15 | 7 | 1 | 1 | 11 | 11 | Only when stainless-steel welding |
| Paint pyrolysis fumes | 23 | 6 | 12 | 5 | 6 | 16 | 1 | 1 | 12 | 10 | Welding on painted steel |
| *Copper fumes | 22 |  | 3 | 19 | 3 | 18 | 1 | 12 | 5 | 5 | Copper fumes during welding on galvanized steel and using bronze rods. |
| *Zinc fumes | 22 | 1 | 4 | 17 | 1 | 10 | 11 | 11 | 10 | 1 | Welding galvanized steel |
| Nickel | 22 | 4 | 4 | 14 | 16 | 5 | 1 | 1 | 10 | 11 | Only when stainless-steel welding |
| Iron oxides | 21 |  | 16 | 5 | 14 | 5 | 2 | 11 | 5 | 5 | Very common in construction industry. Found as rust on mild steel. Cleaning the surface with abrasives. |
| Environmental tobacco smoke | 21 | 4 | 3 | 14 | 16 | 5 |  |  |  | 21 |  |
| Stainless steel dust | 19 | 6 | 4 | 9 | 7 | 6 | 6 | 7 | 8 | 4 | If Stainless-steel is the work piece, Grinding to smooth the weld or at the time of finishing |
| Chromium (VI) | 16 | 3 | 3 | 10 | 13 | 3 |  | 1 | 7 | 8 | Welding stainless steel. |
| Zinc | 15 | 5 | 3 | 7 | 11 | 4 |  | 3 | 8 | 4 | Welding galvanized steel |
| *Tin fumes | 14 |  | 6 | 8 | 3 | 11 |  | 8 | 5 | 1 | Tin/Lead solder |
| *Aluminum fumes | 13 |  |  | 13 |  | 10 | 3 | 5 | 6 | 2 |  |
| Amphibole asbestos | 12 | 10 | 2 |  | 9 | 3 |  | 2 | 5 | 5 | Slag from electrodes and gloves |
| *Lead fumes | 11 | 1 | 5 | 5 | 3 | 6 | 2 | 7 | 4 |  |  |
| Mineral spirits post 1970 | 11 | 2 | 2 | 7 | 4 | 7 |  | 1 | 9 | 1 | Commonly used to clean metal parts before welding |
| Mineral spirits pre 1970 | 11 | 1 | 2 | 8 | 2 | 9 |  |  | 10 | 1 | Commonly used to clean metal parts before welding |
| Copper | 11 | 2 | 2 | 7 | 8 | 3 |  | 3 | 6 | 2 | Copper fumes during welding on galvanized steel and using bronze rods. |
| Tin | 11 | 2 | 2 | 7 | 8 | 3 |  | 4 | 7 |  | Tin/Lead soldering |
| Sulphuric acid | 10 |  | 1 | 9 | 5 | 5 |  | 4 | 4 | 2 | Commonly used acid for cleaning metals before welding |
| Lead | 10 | 3 | 5 | 2 | 6 | 4 |  | 3 | 5 | 2 | Tin/Lead soldering, welding on painted surfaces |
| *Aluminum alloy dust | 9 |  | 2 | 7 | 2 | 7 |  | 2 | 5 | 2 | Dust from grinding Aluminum |
| Strong inorganic acid mist | 9 | 4 |  | 5 | 6 | 3 |  | 8 | 1 |  | metal degreasing before welding |
| Inorganic insulation dust | 8 |  | 5 | 3 | 3 | 5 |  | 4 | 3 | 1 | Heat insulators including asbestos (chrysotile or amphibole), mineral wool, fiberglass. |
| Aluminum | 8 | 1 | 1 | 6 | 4 | 4 |  | 3 | 2 | 3 | Dust from grinding and fumes from welding Aluminium |
| Diesel engine emissions (any) | 6 | 1 | 3 | 2 | 5 |  | 1 | 1 | 4 | 1 | Diesel operated lift trucks |
| Metal coatings | 6 | 4 | 1 | 1 | 5 | 1 |  |  | 2 | 4 |  |
| Diesel engine emissions (light) | 6 | 1 | 3 | 2 | 5 |  | 1 | 1 | 4 | 1 | Diesel operated lift trucks |
| Mineral wool fibers | 5 | 4 | 1 |  | 1 | 4 |  | 3 | 2 |  |  |
| *Zinc dust | 5 |  | 1 | 4 | 5 |  |  | 1 | 4 |  |  |
| Sulphur dioxide | 5 | 2 | 1 | 2 | 3 | 2 |  |  |  | 5 |  |
| Soldering fumes | 5 | 1 | 1 | 3 |  | 5 |  | 2 | 1 | 2 |  |
| Cleaning agents | 5 |  |  | 5 | 4 | 1 |  |  | 5 |  |  |
| Cellulose | 4 |  |  | 4 | 3 |  | 1 |  |  | 4 |  |
| Hydrogen sulphide | 4 | 2 |  | 2 | 2 | 2 |  |  |  | 4 |  |
| Propane | 4 |  | 1 | 3 | 1 | 3 |  |  | 2 | 2 |  |
| Leaded engine emissions | 4 |  | 2 | 2 | 3 | 1 |  |  | 3 | 1 |  |
| Propane combustion products | 4 | 1 | 1 | 2 | 4 |  |  | 2 | 2 |  |  |
| Alkali, caustic solutions | 4 |  | 2 | 2 | 3 | 1 |  | 1 |  | 3 |  |
| Glass fibers | 4 | 4 |  |  |  | 4 |  | 2 | 2 |  |  |
| Bronze dust | 3 |  |  | 3 | 2 | 1 |  | 3 |  |  |  |
| Sulfur | 3 | 2 |  | 1 |  | 3 |  | 2 |  | 1 |  |
| *Copper dust | 3 |  |  | 3 | 1 | 2 |  | 3 |  |  |  |
| Chlorine | 3 | 3 |  |  | 3 |  |  |  |  | 3 |  |
| Chlorine dioxide | 3 | 3 |  |  | 3 |  |  |  |  | 3 |  |
| *Silver fumes | 3 |  | 1 | 2 | 1 | 2 |  | 1 | 2 |  |  |
| Propane engine emissions | 3 |  |  | 3 | 3 |  |  |  |  | 3 |  |
| Leaded gasoline | 3 |  |  | 3 |  | 3 |  | 1 | 1 | 1 |  |
| Lubricating oils and greases (mineral-based) | 3 |  |  | 3 | 2 | 1 |  |  | 2 | 1 |  |
| Hypochlorites | 3 | 2 | 1 |  | 3 |  |  |  |  | 3 |  |
| Cadmium | 3 | 1 | 1 | 1 |  | 3 |  | 2 | 1 |  |  |
| Ionizing radiation | 3 |  | 3 |  | 3 |  |  | 3 |  |  |  |
| Bleaches | 3 |  | 1 | 2 | 3 |  |  |  |  | 3 |  |
| *Mineral-based oils and greases | 3 |  |  | 3 | 1 | 2 |  |  | 2 | 1 |  |
| Brass dust | 2 |  |  | 2 | 2 |  |  | 2 |  |  |  |
| Inorganic pigments | 2 |  |  | 2 | 1 | 1 |  | 1 | 1 |  |  |
| *Iron dust | 2 |  |  | 2 |  | 2 |  |  | 1 | 1 |  |
| Hydrogen chloride | 2 |  | 1 | 1 | 2 |  |  | 1 | 1 |  |  |
| Methane | 2 |  |  | 2 |  | 2 |  |  |  | 2 |  |
| Benzene | 2 |  |  | 2 | 2 |  |  |  |  | 2 |  |
| Toluene | 2 |  |  | 2 | 2 |  |  |  |  | 2 |  |
| Xylene | 2 |  |  | 2 | 2 |  |  |  |  | 2 |  |
| Diesel oil (light) | 2 | 2 |  |  |  | 2 |  |  | 2 |  |  |
| Heating oil | 2 | 2 |  |  |  | 2 |  |  | 2 |  |  |
| Crude oil | 2 |  | 2 |  |  | 2 |  |  | 2 |  |  |
| Unleaded gasoline | 2 |  |  | 2 |  | 2 |  | 1 | 1 |  |  |
| Solvent based coatings | 2 | 1 |  | 1 | 2 |  |  |  | 1 | 1 |  |
| Electric fields | 2 |  | 2 |  | 2 |  |  |  | 1 | 1 |  |
| Extenders | 1 |  |  | 1 | 1 |  |  |  | 1 |  |  |
| Calcium sulphate | 1 |  |  | 1 |  |  | 1 |  |  | 1 |  |
| Calcium carbonate | 1 |  |  | 1 | 1 |  |  | 1 |  |  |  |
| *Cadmium dust | 1 |  |  | 1 |  | 1 |  |  | 1 |  |  |
| Wood dust | 1 | 1 |  |  | 1 |  |  | 1 |  |  |  |
| Phosphoric acid | 1 |  |  | 1 |  | 1 |  |  |  | 1 |  |
| Asphalt | 1 |  |  | 1 |  | 1 |  | 1 |  |  |  |
| Hydraulic fluid | 1 |  |  | 1 | 1 |  |  |  |  | 1 |  |
| Cutting fluids post-1955 (straight, mineral-based) | 1 |  |  | 1 | 1 |  |  |  | 1 |  |  |
| Silver | 1 |  |  | 1 | 1 |  |  |  | 1 |  |  |
| Biocides | 1 |  | 1 |  |  | 1 |  |  | 1 |  |  |
| Unleaded engine emissions | 1 |  | 1 |  | 1 |  |  |  | 1 |  |  |
| Diesel engine emissions (heavy) | 1 |  | 1 |  | 1 |  |  |  |  | 1 |  |
| Insecticides | 1 |  |  | 1 |  | 1 |  | 1 |  |  |  |
| Herbicides | 1 |  |  | 1 |  | 1 |  | 1 |  |  |  |
| Microorganisms | 1 |  |  | 1 |  | 1 |  |  | 1 |  |  |

Table S2. List of the 90 blue-collar occupations included in the analysis

| **CCDO code and title** | **Number of jobs** | |
| --- | --- | --- |
|  | **Lung** | **PROtEuS** |
| 3135114: ORDERLY (medical)* | 13 | 56 |
| 4131134: ACCOUNTING CLERK (clerical) | 54 | 43 |
| 4135182: UTILITY CLERK, BANK (bank. & finance) | 14 | 10 |
| 4153118: SHIPPING AND RECEIVING CLERK (clerical) | 39 | 43 |
| 4153126: SHIPPING CLERK (clerical) | 37 | 76 |
| 4155126: STOREKEEPER (clerical) | 23 | 89 |
| 4155146: INVENTORY CLERK (clerical) | 12 | 23 |
| 4172110: LETTER CARRIER (gov. serv.)* | 26 | 32 |
| 4177118: MESSENGER (clerical) | 14 | 15 |
| 4177122: DELIVERY PERSON (ret. trade) | 74 | 104 |
| 4197114: CLERK, GENERAL OFFICE (clerical) | 64 | 78 |
| 5133126: SALES REPRESENTATIVE, COMMERCIAL AND INDUSTRIAL EQUIPMENT AND SUPPLIES (whole. trade)* | 15 | 51 |
| 5133130: SALES REPRESENTATIVE, FOOD PRODUCTS (whole. trade)* | 26 | 22 |
| 5133199: OTHER COMMERCIAL TRAVELLERS* | 15 | 72 |
| 5135110: SALESPERSON, MOTOR VEHICLES (ret. trade)* | 21 | 28 |
| 5135154: SALESPERSON, HARDWARE (ret. trade; whole. trade)* | 13 | 52 |
| 5137111: SUPERMARKET CLERK (ret. trade) | 22 | 54 |
| 5137114: SALES CLERK (ret. trade) | 74 | 99 |
| 5145110: SERVICE-STATION ATTENDANT (motor vehicle; ret. trade)* | 23 | 26 |
| 5172118: SALESPERSON, REAL ESTATE (insur. & real estate) | 26 | 73 |
| 5193118: ROUTE DRIVER (any ind.)* | 70 | 46 |
| 6112158: POLICE OFFICER (gov. serv.)* | 19 | 95 |
| 6115138: SECURITY GUARD (any ind.) | 85 | 106 |
| 6117190: INFANTRY SOLDIER (military)* | 75 | 74 |
| 6121127: COOK, FIRST (cater. & lodg.)* | 25 | 10 |
| 6121130: SHORT-ORDER COOK (cater. & lodg.)* | 13 | 14 |
| 6121134: COOK, THIRD (cater. & lodg.)* | 16 | 24 |
| 6123110: BARTENDER (cater. & lodg.)* | 23 | 28 |
| 6125126: WAITER/WAITRESS (cater. & lodg.)* | 71 | 72 |
| 6165126: PRESSER, MACHINE (garment & fabric; laund., clean. & press.)* | 28 | 52 |
| 6191110: JANITOR (any ind.)* | 87 | 140 |
| 6191114: CLEANER, light DUTY (any ind.)* | 36 | 50 |
| 6191118: CLEANER, INDUSTRIAL-PLANT (any ind.) | 18 | 14 |
| 6198134: KITCHEN HELPER (cater. & lodg.)* | 10 | 16 |
| 6198170: DISHWASHER (cater. & lodg.)* | 15 | 32 |
| 7111110: FARMER, GENERAL (agric.)* | 22 | 37 |
| 7181110: FARM WORKER, GENERAL (agric.)* | 94 | 26 |
| 7183122: FARM WORKER, VEGETABLE (agric.) | 10 | 12 |
| 7195146: LANDSCAPE WORKER (agric.)* | 14 | 15 |
| 7198112: FARM LABOURER, GENERAL (agric.)* | 29 | 28 |
| 7513122: LOGGER, ALL-ROUND (forest. & log.)* | 88 | 58 |
| 8213114: BAKER (bake. prod.; cater. & lodg.; food & bev., n.e.c.)* | 20 | 57 |
| 8215110: BUTCHER, ALL-ROUND (slaught. & meat pack.)* | 26 | 52 |
| 8215114: MEAT CUTTER (ret. trade; slaught. & meat pack.)* | 10 | 18 |
| 8238134: SAWMILL LABOURER (pulp & paper; sawmill; woodworking)* | 12 | 13 |
| 8311110: TOOL AND DIE MAKER (mach., weld. & forg.)* | 14 | 13 |
| 8313154: MACHINIST, GENERAL (mach., weld. & forg.)* | 36 | 106 |
| 8333118: SHEET-METAL WORKER (construction; metal stamp., press. & coat.)* | 19 | 35 |
| 8335126: WELDER, COMBINATION (mach., weld. & forg.)* | 37 | 53 |
| 8335138: WELDER, ARC (mach., weld. & forg.)* | 22 | 59 |
| 8541110: CABINETMAKER (furn.)* | 24 | 60 |
| 8551146: CUTTER, PORTABLE MACHINE (garment & fabric)* | 18 | 29 |
| 8553110: TAILOR, MADE-TO-MEASURE GARMENTS (garment & fabric; ret. trade)* | 18 | 28 |
| 8563114: SEWING-MACHINE OPERATOR (any ind.)* | 28 | 15 |
| 8581110: MOTOR-VEHICLE MECHANIC (motor vehicle)* | 73 | 82 |
| 8581118: INDUSTRIAL-TRUCK MECHANIC (mech. equip., n.e.c.)* | 17 | 19 |
| 8581142: BODY REPAIRER (motor vehicle)* | 21 | 61 |
| 8582110: AIRCRAFT MECHANIC (air & space-craft)* | 15 | 22 |
| 8584122: MILLWRIGHT (mech. equip., n.e.c.)* | 14 | 74 |
| 8711110: HEAVY-DUTY-EQUIPMENT OPERATOR (any ind.)* | 36 | 29 |
| 8733122: ELECTRICIAN (construction)* | 41 | 111 |
| 8781110: CARPENTER (construction)* | 65 | 104 |
| 8781121: CONCRETE FORMER (construction)* | 10 | 18 |
| 8782110: BRICKLAYER (construction)* | 15 | 18 |
| 8785110: PAINTER AND DECORATOR (construction)* | 14 | 24 |
| 8785120: PAINTER (construction)* | 30 | 27 |
| 8787118: ROOFER, ASPHALT (construction)* | 10 | 10 |
| 8791110: PIPE FITTER (construction)* | 14 | 27 |
| 8791114: PLUMBER (construction)* | 14 | 56 |
| 8793114: STRUCTURAL-STEEL ERECTOR (construction)* | 13 | 22 |
| 8798114: CONSTRUCTION LABOURER (construction)* | 148 | 73 |
| 9171110: BUS DRIVER (motor trans.)* | 34 | 102 |
| 9173110: TAXI DRIVER (motor trans.)* | 70 | 105 |
| 9173114: CHAUFFEUR (motor trans.)* | 13 | 31 |
| 9175110: TRUCK DRIVER, GENERAL (motor trans.)* | 130 | 176 |
| 9175114: DRIVER, TANK TRUCK (motor trans.)* | 19 | 26 |
| 9175118: TRUCK DRIVER, HEAVY (motor trans.)* | 65 | 87 |
| 9175122: TRUCK DRIVER, TRACTOR-TRAILER (motor trans.)* | 12 | 15 |
| 9175130: DRIVER, DUMP-TRUCK (motor trans.)* | 22 | 16 |
| 9175138: TRUCK DRIVER, LIGHT (motor trans.)* | 62 | 54 |
| 9179190: TRUCK-DRIVER HELPER (motor trans.)* | 14 | 22 |
| 9313110: LONGSHORE WORKER (water trans.)* | 24 | 20 |
| 9315126: INDUSTRIAL-TRUCK OPERATOR (any ind.)* | 33 | 101 |
| 9317218: PACKAGER, MACHINE (any ind.)* | 14 | 17 |
| 9318110: MATERIAL HANDLER, GENERAL (any ind.)* | 68 | 73 |
| 9318114: MATERIAL HANDLER, HEAVY (any ind.)* | 33 | 22 |
| 9318118: MATERIAL HANDLER, LIGHT (any ind.)* | 33 | 28 |
| 9318122: PACKAGER, HAND (any ind.)* | 35 | 51 |
| 9512126: OFFSET PRESS OPERATOR (print. & pub.)* | 15 | 37 |
| 9918110: LABOURER, MUNICIPAL (gov. serv.)* | 39 | 25 |

* Denotes an occupation with an expert-annotated job-exposure profile. n.e.c.: Not elsewhere classified

Table S3. Proportion of jobs exposed by agent in PROtEuS (n=313), overall and stratified by blue/white-collar status, and proportion of PROtEuS and Lung cancer study jobs exposed among the blue-collar occupations (n=90) and agents (n=203) retained in the comparison

| **Group/agent** | **Global prevalence in PROtEuS (%)** | | | **Prevalence in blue-collar jobs in comparison (%)** | |
| --- | --- | --- | --- | --- | --- |
|  | **All jobs^1^** | **Blue-collar jobs^2^** | **White-collar jobs^3^** | **PROtEuS^4^** | **Lung^5^** |
| **Metals** |  |  |  |  |  |
| Alumina | 7.0 | 10.7 | 2.0 | 13.2 | 8.6 |
| Aluminum | 8.5 | 12.6 | 3.0 |  |  |
| Antimony | 0.2 | 0.4 | 0.0 | 0.0 | 0.1 |
| Arc welding fumes | 5.4 | 7.1 | 3.2 | 8.5 | 7.4 |
| Arsenic | 0.5 | 0.8 | 0.1 | 0.3 | 0.8 |
| Basic lead carbonate | 0.3 | 0.5 | 0.0 | 0.9 | 0.4 |
| Beryllium | 0.1 | 0.0 | 0.1 |  |  |
| Brass dust | 0.4 | 0.6 | 0.2 | 0.6 | 0.7 |
| Bronze dust | 0.4 | 0.6 | 0.1 | 0.9 | 0.9 |
| Cadmium | 1.9 | 2.7 | 0.8 |  |  |
| Chromium | 4.4 | 6.2 | 1.9 |  |  |
| Chromium (VI) | 2.7 | 4.0 | 0.9 |  |  |
| Cobalt | 0.2 | 0.2 | 0.1 | 0.2 | 0.1 |
| Copper | 3.9 | 5.9 | 1.1 |  |  |
| Gas welding fumes | 5.2 | 7.2 | 2.5 | 8.2 | 6.9 |
| Gold compounds | 0.0 | 0.0 | 0.0 |  |  |
| Inorganic pigments | 5.1 | 6.9 | 2.7 | 6.4 | 7.2 |
| Iron | 11.6 | 16.3 | 5.2 |  |  |
| Iron oxides | 3.1 | 4.8 | 0.8 | 6.0 | 5.8 |
| Lead | 8.4 | 12.5 | 2.9 |  |  |
| Lead chromate | 1.1 | 1.8 | 0.3 | 2.8 | 1.3 |
| Lead oxides | 0.5 | 0.7 | 0.1 | 0.9 | 0.3 |
| Magnesium | 0.1 | 0.2 | 0.0 |  |  |
| Manganese | 4.4 | 6.4 | 1.6 |  |  |
| Mercury | 0.4 | 0.3 | 0.6 |  |  |
| Metal oxide fumes | 9.3 | 12.4 | 5.1 | 12.9 | 9.9 |
| Metallic dust | 13.8 | 18.3 | 7.6 | 17.5 | 12.6 |
| Mild steel dust | 8.9 | 12.7 | 3.8 | 13.0 | 10.6 |
| Nickel | 2.5 | 3.5 | 1.2 |  |  |
| Selenium | 0.6 | 0.9 | 0.1 | 1.5 | 1.9 |
| Silver | 0.8 | 0.9 | 0.7 |  |  |
| Soldering fumes | 4.3 | 5.7 | 2.4 | 6.0 | 3.3 |
| Stainless steel dust | 1.9 | 2.7 | 0.8 | 3.3 | 3.5 |
| Tin | 3.5 | 4.6 | 1.9 |  |  |
| Titanium | 1.2 | 1.9 | 0.3 |  |  |
| Titanium dioxide | 0.8 | 1.3 | 0.2 | 2.3 | 3.0 |
| Tungsten compounds | 0.2 | 0.4 | 0.0 |  |  |
| Vanadium | 0.0 | 0.0 | 0.0 |  |  |
| Zinc | 2.9 | 4.3 | 1.0 |  |  |
| Zinc oxide | 0.2 | 0.3 | 0.0 | 0.3 | 0.2 |
| **Organic Solvents** | |  |  |  |  |
| 1,1,1-Trichlorethane | 0.4 | 0.6 | 0.2 | 0.7 | 0.5 |
| Acetone | 1.5 | 1.3 | 1.7 | 0.9 | 0.6 |
| Aliphatic alcohols | 7.9 | 8.8 | 6.7 |  |  |
| Aliphatic esters | 0.8 | 1.1 | 0.5 |  |  |
| Aliphatic ketones | 2.4 | 2.7 | 2.0 |  |  |
| Aromatic alcohols | 0.8 | 1.0 | 0.6 |  |  |
| Benzene | 4.7 | 6.8 | 1.8 |  |  |
| Carbon tetrachloride | 0.6 | 0.9 | 0.2 | 0.2 | 0.2 |
| Chlorinated alkanes | 2.0 | 2.7 | 1.1 |  |  |
| Chlorinated alkenes | 1.6 | 2.2 | 0.9 |  |  |
| Chloroform | 0.2 | 0.1 | 0.3 |  |  |
| Diethyl ether | 0.2 | 0.1 | 0.3 | 0.1 | 0.1 |
| Ethanol | 0.6 | 0.6 | 0.7 | 0.2 | 0.5 |
| Ethylene glycol | 2.3 | 3.1 | 1.1 | 4.1 | 3.8 |
| Glycol ethers | 0.2 | 0.3 | 0.2 | 0.3 | 0.3 |
| Isopropanol | 4.0 | 5.3 | 2.2 | 7.6 | 7.4 |
| Methanol | 2.4 | 2.0 | 3.0 | 2.7 | 4.8 |
| Methyl ethyl ketone | 0.0 | 0.0 | 0.0 |  |  |
| Methylene chloride | 0.4 | 0.6 | 0.2 | 0.4 | 0.8 |
| Mineral spirits post 1970 | 8.3 | 11.7 | 3.8 | 13.2 | 7.0 |
| Mineral spirits pre 1970 | 5.4 | 8.5 | 1.3 | 8.6 | 8.1 |
| Mononuclear aromatic hydrocarbons | 14.5 | 21.1 | 5.7 |  |  |
| n-Hexane | 1.4 | 1.9 | 0.7 | 2.8 | 1.7 |
| Organic Solvents | 22.7 | 28.3 | 15.3 | 27.4 | 25.9 |
| Perchloroethylene | 0.2 | 0.4 | 0.1 | 0.3 | 0.5 |
| Styrene | 1.0 | 1.6 | 0.1 | 1.5 | 0.7 |
| Toluene | 4.9 | 6.8 | 2.2 |  |  |
| Trichloroethylene | 0.5 | 0.6 | 0.3 | 0.7 | 0.5 |
| Xylene | 4.0 | 5.7 | 1.6 |  |  |
| **Polycyclic aromatic hydrocarbons** | | |  |  |  |
| Benzo[a]pyrene | 4.9 | 7.5 | 1.4 |  |  |
| PAHs from any source | 19.1 | 27.6 | 7.5 |  |  |
| PAHs from coal | 1.5 | 2.1 | 0.6 |  |  |
| PAHs from other sources | 3.5 | 5.3 | 1.2 |  |  |
| PAHs from petroleum | 16.3 | 23.8 | 6.2 |  |  |
| PAHs from wood | 1.5 | 2.4 | 0.2 |  |  |
| **Paints, varnishes and inks** | | |  |  |  |
| Artistic paints | 0.1 | 0.0 | 0.3 |  |  |
| Gypsum / Plaster coatings | 1.8 | 2.3 | 1.2 | 3.7 | 4.5 |
| Inks | 3.2 | 2.4 | 4.2 | 1.2 | 1.3 |
| Metal coatings | 2.2 | 3.1 | 1.0 | 3.1 | 3.4 |
| Other paints,varnishes | 0.0 | 0.0 | 0.0 |  |  |
| Solvent based coatings | 4.4 | 6.1 | 2.2 | 6.8 | 7.4 |
| Water based coatings | 1.7 | 1.9 | 1.4 | 3.0 | 3.3 |
| Wood paints | 1.1 | 1.6 | 0.5 | 2.0 | 2.4 |
| Wood stains | 0.7 | 1.1 | 0.2 | 1.5 | 1.4 |
| Wood varnishes | 1.1 | 1.5 | 0.5 | 1.9 | 1.8 |
| Wood varnishes, stains | 0.0 | 0.0 | 0.0 |  |  |
| **Combustion products and engine emissions** | | | |  |  |
| Ashes | 1.6 | 2.5 | 0.4 | 2.8 | 4.6 |
| Coal combustion products | 0.6 | 0.9 | 0.2 | 0.6 | 1.2 |
| Coke combustion products | 0.0 | 0.1 | 0.0 | 0.0 | 0.1 |
| Cooking fumes | 3.5 | 5.1 | 1.3 | 5.0 | 5.2 |
| Diesel engine emissions | 17.7 | 23.3 | 10.2 | 31.4 | 31.0 |
| Diesel engine emissions (heavy) | 0.6 | 0.7 | 0.4 | 0.0 | 0.4 |
| Diesel engine emissions (light) | 17.6 | 23.2 | 10.1 | 31.4 | 30.9 |
| Jet fuel engine emissions | 0.8 | 0.5 | 1.2 | 0.5 | 0.3 |
| Jet fuel engine emissions (wide-cut) | 0.1 | 0.0 | 0.1 |  |  |
| Leaded engine emissions | 16.1 | 21.3 | 9.1 | 29.3 | 42.3 |
| Liquid fuel combustion products | 1.6 | 2.5 | 0.5 | 2.1 | 2.4 |
| Mineral-based oil & grease pyrolysis fumes | 5.2 | 7.9 | 1.6 | 8.2 | 6.2 |
| Natural gas combustion products | 2.9 | 3.9 | 1.5 | 3.4 | 2.5 |
| Other pyrolysis fumes | 0.0 | 0.0 | 0.0 |  |  |
| Paint pyrolysis fumes | 2.0 | 3.1 | 0.5 | 3.7 | 4.0 |
| Plastics pyrolysis fumes | 1.2 | 1.6 | 0.7 | 0.5 | 1.1 |
| Propane combustion products | 3.4 | 4.7 | 1.5 | 5.1 | 3.6 |
| Propane engine emissions | 2.0 | 2.8 | 1.0 | 3.3 | 5.0 |
| Rubber pyrolysis fumes | 1.2 | 1.9 | 0.3 | 1.1 | 0.6 |
| Unleaded engine emissions | 16.7 | 17.6 | 15.5 | 23.5 | 14.2 |
| Wood combustion products | 1.4 | 2.4 | 0.2 | 2.3 | 3.4 |
| **Petroleum products** | |  |  |  |  |
| Alkanes (C18+) | 16.2 | 23.6 | 6.1 |  |  |
| Alkanes (C5-C17) | 26.7 | 34.6 | 16.1 |  |  |
| Asphalt | 1.1 | 1.4 | 0.7 | 2.1 | 2.9 |
| Aviation gasoline | 0.3 | 0.3 | 0.3 | 0.4 | 0.3 |
| Bunker C heating oil | 0.0 | 0.1 | 0.0 |  |  |
| Crude petroleum | 0.2 | 0.2 | 0.2 | 0.1 | 0.2 |
| Diesel oil | 3.2 | 5.2 | 0.4 | 7.2 | 4.0 |
| Heating oil | 0.9 | 1.3 | 0.3 | 1.3 | 1.3 |
| Jet fuel (JP5, Jet A, Jet A1) | 0.4 | 0.4 | 0.4 | 0.3 | 0.2 |
| Jet fuel (wide-cut) (JP4, Jet B) | 0.0 | 0.0 | 0.0 |  |  |
| Kerosene | 0.5 | 0.8 | 0.1 | 1.3 | 2.5 |
| Leaded gasoline | 6.6 | 8.7 | 3.9 | 11.6 | 16.1 |
| Other mineral oils | 0.7 | 1.0 | 0.2 | 1.0 | 0.7 |
| Paraffin | 0.0 | 0.0 | 0.0 |  |  |
| Textile oils | 0.4 | 0.7 | 0.0 | 0.0 | 0.1 |
| Unleaded gasoline | 8.9 | 9.2 | 8.5 | 12.0 | 5.6 |
| **Carbon dust** | |  |  |  |  |
| Carbon black | 1.6 | 1.5 | 1.8 | 1.0 | 1.1 |
| Coal dust | 0.3 | 0.4 | 0.1 | 0.3 | 0.8 |
| Coke dust | 0.0 | 0.1 | 0.0 | 0.0 | 0.2 |
| Graphite dust | 0.2 | 0.3 | 0.1 |  |  |
| **Soot** |  |  |  |  |  |
| Coal soot | 0.1 | 0.1 | 0.0 |  |  |
| Petroleum soot | 1.9 | 2.9 | 0.5 |  |  |
| Soot from any source | 2.5 | 4.0 | 0.5 |  |  |
| Wood Soot | 0.5 | 0.8 | 0.0 | 0.1 | 0.2 |
| **Cutting fluids** | |  |  |  |  |
| Cutting fluids | 0.1 | 0.1 | 0.1 |  |  |
| Cutting fluids (emulsified, mineral-based) | 1.5 | 2.2 | 0.5 | 2.9 | 1.1 |
| Cutting fluids (synthetic) | 0.0 | 0.1 | 0.0 | 0.1 | 0.0 |
| Cutting fluids post-1955 (straight, mineral-based) | 2.1 | 2.9 | 1.1 | 3.8 | 2.7 |
| Cutting fluids pre-1955 (straight, mineral-based) | 0.1 | 0.2 | 0.1 | 0.1 | 1.0 |
| **Synthetic oils and greases** | | |  |  |  |
| Silicone oils and greases | 0.0 | 0.1 | 0.0 |  |  |
| Synthetic oils and greases | 0.0 | 0.0 | 0.0 |  |  |
| **Complex organic fluids** | |  |  |  |  |
| Animal, vegetable glues | 0.2 | 0.3 | 0.0 | 0.1 | 0.4 |
| Coal tar and pitch | 0.6 | 0.7 | 0.4 | 1.0 | 1.3 |
| Heat transfer oils | 0.0 | 0.0 | 0.0 |  |  |
| Hydraulic fluid | 3.0 | 4.5 | 0.9 | 4.4 | 4.2 |
| Ink formulation oils | 0.6 | 0.8 | 0.4 | 0.8 | 0.7 |
| Linseed oil | 1.1 | 1.6 | 0.4 | 2.1 | 2.6 |
| Lubricating oils and greases (mineral-based) | 10.8 | 16.3 | 3.5 | 16.4 | 11.9 |
| Lubricating oils and greases (synthetic) | 0.1 | 0.1 | 0.1 |  |  |
| Polyvinyl Acetate | 0.0 | 0.0 | 0.0 |  |  |
| Synthetic adhesives | 5.9 | 8.0 | 3.0 | 7.8 | 5.9 |
| Waxes, polishes | 1.7 | 2.6 | 0.3 | 3.1 | 2.5 |
| **Gases** |  |  |  |  |  |
| Acetylene | 2.6 | 3.9 | 0.9 | 4.8 | 3.3 |
| Aliphatic aldehydes | 7.4 | 10.1 | 3.8 |  |  |
| Alkanes (C1-C4) | 3.7 | 5.2 | 1.6 |  |  |
| Ammonia | 5.0 | 6.2 | 3.4 |  |  |
| Anaesthetic gases | 0.4 | 0.3 | 0.6 | 0.3 | 0.0 |
| Carbon monoxide | 11.1 | 16.3 | 4.0 |  |  |
| Chlorine | 0.8 | 1.0 | 0.5 | 0.1 | 0.3 |
| Chlorine dioxide | 0.2 | 0.2 | 0.3 | 0.0 | 0.4 |
| Coal gas | 0.0 | 0.0 | 0.0 |  |  |
| Ethylene | 0.0 | 0.0 | 0.0 |  |  |
| Ethylene oxide | 0.1 | 0.0 | 0.1 |  |  |
| Fluorocarbons | 1.2 | 1.8 | 0.3 |  |  |
| Formaldehyde | 6.3 | 8.7 | 2.9 | 7.7 | 10.0 |
| Hydrogen | 0.1 | 0.2 | 0.0 |  |  |
| Hydrogen cyanide | 0.6 | 1.0 | 0.1 |  |  |
| Methane | 1.5 | 2.0 | 0.7 |  |  |
| Natural gas | 0.4 | 0.4 | 0.4 | 0.3 | 0.3 |
| Nitrogen oxides | 8.9 | 13.2 | 3.1 |  |  |
| Ozone | 5.9 | 6.5 | 5.1 | 7.9 | 6.3 |
| Phosgene | 0.5 | 0.9 | 0.0 | 0.2 | 0.1 |
| Propane | 1.5 | 2.0 | 0.8 | 2.2 | 1.9 |
| Propellant gases | 1.7 | 2.6 | 0.7 | 1.4 | 1.0 |
| Sulphur dioxide | 2.7 | 3.5 | 1.7 |  |  |
| Trichlorotrifluoroethane or CFC-113 | 0.2 | 0.3 | 0.1 | 0.2 | 0.1 |
| Unsaturated aliphatic hydrocarbons | 2.9 | 4.1 | 1.2 |  |  |
| Vinyl chloride | 0.3 | 0.5 | 0.1 | 0.1 | 0.2 |
| **Acids and bases** | |  |  |  |  |
| Acetic acid | 0.9 | 0.8 | 1.0 | 0.3 | 0.7 |
| Acrylonitrile | 0.0 | 0.0 | 0.0 |  |  |
| All acids | 0.1 | 0.0 | 0.1 |  |  |
| Carbon disulphide | 0.1 | 0.1 | 0.1 |  |  |
| Caustic alkali solutions | 2.7 | 3.3 | 1.9 | 3.0 | 2.0 |
| Cyanides | 0.8 | 1.2 | 0.2 |  |  |
| Fluorides | 1.0 | 1.4 | 0.4 |  |  |
| Formic acid | 0.1 | 0.1 | 0.1 | 0.0 | 0.1 |
| Hydrogen chloride | 3.8 | 4.7 | 2.5 | 4.2 | 4.0 |
| Hydrogen fluoride | 0.9 | 1.3 | 0.3 | 1.6 | 2.5 |
| Hydrogen peroxide | 0.5 | 0.3 | 0.6 |  |  |
| Hydrogen sulphide | 1.7 | 2.5 | 0.7 |  |  |
| Hypochlorites | 2.8 | 4.0 | 1.1 | 5.7 | 2.7 |
| Inorganic acid solutions | 4.7 | 6.1 | 2.7 | 6.5 | 7.2 |
| Javel water | 0.0 | 0.0 | 0.0 |  |  |
| Methyl methacrylate | 0.1 | 0.0 | 0.3 | 0.0 | 0.0 |
| Nitrates | 0.0 | 0.0 | 0.0 | 0.0 | 0.1 |
| Nitric acid | 0.6 | 0.5 | 0.6 | 0.0 | 0.1 |
| Phenol | 0.1 | 0.1 | 0.2 | 0.0 | 0.2 |
| Phosphoric acid | 0.4 | 0.5 | 0.3 | 0.7 | 0.7 |
| Plating solutions | 0.3 | 0.4 | 0.1 | 0.2 | 0.1 |
| Sulphuric acid | 3.1 | 3.8 | 2.2 | 3.1 | 4.4 |
| Turpentine | 0.7 | 0.9 | 0.3 | 1.3 | 1.6 |
| Volatile Organic Liquids | 38.7 | 46.7 | 27.8 |  |  |
| **Other organic products** | | |  |  |  |
| Alkyds | 1.3 | 2.0 | 0.4 | 2.7 | 3.2 |
| Aromatic amines | 1.4 | 2.0 | 0.6 | 2.2 | 2.4 |
| Calcium oxide fumes | 0.7 | 1.1 | 0.1 | 1.4 | 2.3 |
| Epichlorohydrin | 0.0 | 0.0 | 0.0 |  |  |
| Glycerine | 0.0 | 0.0 | 0.0 |  |  |
| Isocyanates | 0.6 | 1.0 | 0.2 | 1.0 | 0.4 |
| Nitroglycerine | 0.0 | 0.0 | 0.0 |  |  |
| Organic Sulfur Compounds | 0.0 | 0.0 | 0.0 |  |  |
| Phthalates | 0.3 | 0.5 | 0.1 | 0.7 | 0.5 |
| Polychlorinated biphenyls or PCBs | 0.4 | 0.5 | 0.2 | 0.4 | 0.3 |
| RDX | 0.0 | 0.0 | 0.0 |  |  |
| Trinitrotoluene | 0.0 | 0.0 | 0.0 |  |  |
| **Organic dusts** | |  |  |  |  |
| ABS (acrylonitrile-butadiene-styrene) | 0.2 | 0.3 | 0.0 | 0.6 | 0.2 |
| Acetate fibres | 0.2 | 0.3 | 0.0 | 0.2 | 0.5 |
| Acrylic fibres | 0.5 | 0.8 | 0.2 | 0.5 | 0.7 |
| Cellulose | 6.4 | 8.9 | 3.0 | 10.5 | 9.5 |
| Cellulose acetate | 0.1 | 0.2 | 0.0 | 0.2 | 0.3 |
| Cellulose nitrate | 0.3 | 0.5 | 0.0 | 0.4 | 0.2 |
| Cork dust | 0.1 | 0.2 | 0.0 | 0.0 | 0.1 |
| Cotton dust | 3.7 | 5.7 | 1.1 | 4.9 | 5.1 |
| Epoxies | 0.9 | 1.3 | 0.5 | 1.9 | 0.7 |
| Felt dust | 0.0 | 0.0 | 0.0 | 0.0 | 0.1 |
| Flax fibres | 0.4 | 0.6 | 0.1 | 0.6 | 0.9 |
| Flour dust | 1.2 | 1.9 | 0.3 | 2.3 | 2.8 |
| Fur dust | 0.2 | 0.3 | 0.0 | 0.1 | 0.1 |
| Grain dust | 1.2 | 1.9 | 0.3 | 2.7 | 5.3 |
| Hair dust | 0.3 | 0.5 | 0.0 |  |  |
| Leather dust | 0.8 | 1.2 | 0.2 | 0.3 | 0.2 |
| Melamine-formaldehyde | 0.1 | 0.2 | 0.1 | 0.2 | 0.1 |
| Natural rubber | 0.2 | 0.3 | 0.0 | 0.0 | 0.2 |
| Nylon fibres | 1.2 | 1.8 | 0.5 | 1.5 | 1.7 |
| Organic dyes and pigments | 2.3 | 3.1 | 1.1 | 3.0 | 3.1 |
| Phenol-formaldehyde | 0.1 | 0.2 | 0.1 | 0.1 | 0.2 |
| Plastic dusts | 3.0 | 4.2 | 1.4 | 5.0 | 2.4 |
| Poly(vinyl acetate) | 0.7 | 1.1 | 0.1 | 1.3 | 2.3 |
| Poly(vinyl chloride) | 0.5 | 0.7 | 0.2 | 0.4 | 0.5 |
| Polyacrylates | 1.0 | 1.2 | 0.6 | 1.6 | 1.5 |
| Polyamides | 0.0 | 0.1 | 0.0 | 0.1 | 0.0 |
| Polychloroprene | 0.0 | 0.0 | 0.0 |  |  |
| Polyester fibres | 2.2 | 3.3 | 0.7 | 2.8 | 2.5 |
| Polyesters | 0.5 | 0.8 | 0.1 | 1.5 | 0.6 |
| Polyethylene | 0.1 | 0.1 | 0.1 |  |  |
| Polypropylene | 0.1 | 0.1 | 0.0 |  |  |
| Polystyrene | 0.4 | 0.6 | 0.2 | 0.6 | 0.4 |
| Polyurethanes | 1.1 | 1.5 | 0.5 | 1.9 | 1.3 |
| PVC | 0.0 | 0.0 | 0.0 |  |  |
| PVC dust | 0.0 | 0.0 | 0.0 |  |  |
| Rayon fibres | 0.7 | 1.1 | 0.2 | 1.0 | 1.1 |
| Rosin | 0.1 | 0.0 | 0.1 |  |  |
| Rubber dust | 1.1 | 1.6 | 0.4 | 1.5 | 1.2 |
| Silk fibres | 0.4 | 0.6 | 0.1 | 0.8 | 1.1 |
| Starch dust | 1.6 | 2.5 | 0.3 | 2.1 | 2.0 |
| Styrene-butadiene rubber | 0.2 | 0.4 | 0.1 | 0.1 | 0.2 |
| Sugar dust | 0.7 | 1.1 | 0.1 | 1.5 | 2.0 |
| Synthetic fibres | 3.1 | 4.6 | 1.0 |  |  |
| Tannic acid | 0.1 | 0.1 | 0.0 |  |  |
| Tobacco dust | 0.1 | 0.2 | 0.0 | 0.2 | 0.3 |
| Treated textile fibres | 4.0 | 6.0 | 1.3 | 5.9 | 5.8 |
| Untreated textile fibres | 0.5 | 0.9 | 0.1 | 0.1 | 0.5 |
| Urea-formaldehyde | 0.6 | 0.9 | 0.2 | 1.6 | 0.2 |
| Wood dust | 9.2 | 12.6 | 4.5 | 15.1 | 19.1 |
| Wool fibres | 1.6 | 2.5 | 0.4 | 2.3 | 3.0 |
| **Inorganic dusts** | |  |  |  |  |
| Abrasives dust | 9.5 | 13.9 | 3.6 | 16.3 | 11.7 |
| Amphibole asbestos | 1.6 | 2.5 | 0.4 | 2.2 | 3.2 |
| Brick dust | 1.8 | 2.7 | 0.6 | 2.4 | 2.9 |
| Calcium carbonate | 8.1 | 5.7 | 11.4 | 7.3 | 9.0 |
| Calcium oxide | 1.9 | 2.9 | 0.4 | 3.0 | 3.9 |
| Calcium sulphate | 7.4 | 6.4 | 8.7 | 7.5 | 8.0 |
| Chrysotile asbestos | 6.8 | 10.4 | 2.0 | 10.1 | 10.5 |
| Clay dust | 0.4 | 0.7 | 0.1 | 0.8 | 2.0 |
| Concrete dust | 6.6 | 8.8 | 3.7 | 9.6 | 8.4 |
| Construction site dust | 0.0 | 0.0 | 0.1 |  |  |
| Cosmetic talc | 1.1 | 0.9 | 1.4 | 1.2 | 0.3 |
| Cristalline silica | 7.2 | 9.4 | 4.1 |  |  |
| Extenders | 2.5 | 3.7 | 0.9 | 4.9 | 5.0 |
| Glass dust | 0.3 | 0.5 | 0.1 | 0.2 | 0.3 |
| Glass fibres | 2.0 | 3.0 | 0.5 | 3.3 | 2.4 |
| Industrial talc | 0.8 | 1.3 | 0.2 | 1.7 | 2.5 |
| Inorganic insulation dust | 5.2 | 7.5 | 2.2 | 7.3 | 6.8 |
| Mica | 0.1 | 0.2 | 0.0 | 0.0 | 0.4 |
| Mineral wool fibres | 2.7 | 3.8 | 1.1 | 4.3 | 5.2 |
| Perlite, vermiculite | 0.1 | 0.1 | 0.0 | 0.2 | 0.1 |
| Phosphorus | 0.0 | 0.0 | 0.0 |  |  |
| Portland cement | 3.5 | 4.3 | 2.3 | 5.5 | 5.9 |
| Refractory brick dust | 0.1 | 0.1 | 0.0 | 0.1 | 0.4 |
| Silicates | 0.0 | 0.0 | 0.0 |  |  |
| Silicon carbide | 3.4 | 5.4 | 0.6 | 7.9 | 5.9 |
| Sodium carbonate | 0.4 | 0.6 | 0.2 | 0.2 | 1.3 |
| Sodium hydrosulphite | 0.4 | 0.4 | 0.4 |  |  |
| Soil dust | 12.6 | 16.2 | 7.8 | 21.1 | 19.7 |
| Sulfur | 0.2 | 0.3 | 0.1 | 0.1 | 0.5 |
| Tungsten carbide | 0.2 | 0.4 | 0.1 | 0.5 | 0.4 |
| **Radiation, electric and magnetic fields** | | | |  |  |
| Ionizing radiation | 1.0 | 0.6 | 1.6 | 0.3 | 0.4 |
| Ultraviolet radiation | 11.3 | 16.0 | 5.0 | 20.6 | 27.0 |
| **Pesticides** |  |  |  |  |  |
| Creosote | 0.4 | 0.7 | 0.1 | 0.8 | 0.6 |
| DDT | 0.2 | 0.2 | 0.1 | 0.2 | 0.7 |
| Dichlorobenzene | 0.0 | 0.0 | 0.0 |  |  |
| Fungicides | 0.1 | 0.1 | 0.0 | 0.2 | 0.0 |
| Herbicides | 0.1 | 0.3 | 0.0 | 0.3 | 0.7 |
| Insecticides | 1.8 | 2.7 | 0.5 |  |  |
| Pentachlorophenol | 0.0 | 0.1 | 0.0 |  |  |
| Pesticides | 2.1 | 3.1 | 0.7 |  |  |
| Wood preservatives | 0.3 | 0.6 | 0.0 | 0.5 | 0.4 |
| **General categories** | |  |  |  |  |
| Animal Fat | 0.0 | 0.0 | 0.0 |  |  |
| Antineoplastic medication | 0.0 | 0.0 | 0.0 |  |  |
| Biocides | 5.3 | 6.9 | 3.3 | 8.9 | 10.6 |
| Bleaches | 2.1 | 3.0 | 0.9 | 4.0 | 0.7 |
| Cleaning agents | 12.6 | 19.3 | 3.6 | 22.7 | 17.8 |
| Cosmetics | 0.0 | 0.0 | 0.0 |  |  |
| Fertilizers | 0.8 | 1.4 | 0.1 | 1.9 | 2.6 |
| Laboratory products | 0.0 | 0.0 | 0.0 |  |  |
| Microorganisms | 8.2 | 11.3 | 4.1 | 16.0 | 14.7 |
| Pharmaceuticals | 0.2 | 0.2 | 0.4 |  |  |
| Photographic products | 0.3 | 0.2 | 0.5 |  |  |

1. Percentage of all jobs in PROtEuS (n=16,065) exposed by agent
2. Percentage of all blue-collar jobs in PROtEuS (n=9239) exposed by agent
3. Percentage of all white-collar jobs in PROtEuS (n=6826) exposed by agent
4. Percentage of jobs in PROtEuS retained in the comparison (n=4318) exposed by agent
5. Percentage of Lung cancer study jobs retained in the comparison (n=3022) exposed by agent

Table S4. Cumulative odds ratios and 95% confidence intervals for the association between the categorical confidence, concentration or frequency of exposed and source of exposure data

|  | **N Lung (1)** | **N PROtEuS (2)** | **Confidence  OR (95% CI) (3)** | **Concentration OR (95% CI)** | **Frequency  OR (95% CI)** |
| --- | --- | --- | --- | --- | --- |
| **Overall** | 18864 | 29551 | **1.31 (1.26-1.36)** | **0.92 (0.88-0.95)** | **0.88 (0.85-0.91)** |
| **Occupations with expert-annotated JEPs (n=75)** | 18089 | 28210 | **1.32 (1.27-1.37)** | **0.93 (0.89-0.97)** | **0.90 (0.87-0.93)** |
| **Chemical group (4)** |  |  |  |  |  |
| Metals (n=19) | 2181 | 4223 | 1.09 (0.99-1.21) | 1.09 (0.98-1.21) | **1.13 (1.02-1.25)** |
| Organic Solvents (n=17) | 1590 | 2782 | **1.30 (1.14-1.47)** | **1.85 (1.63-2.09)** | **1.19 (1.06-1.34)** |
| Paints, varnishes and inks (n=8) | 619 | 910 | 1.17 (0.93-1.48) | **0.80 (0.65-0.98)** | 1.11 (0.92-1.34) |
| Combustion products and engine emissions (n=16) | 4412 | 6101 | **1.36 (1.26-1.46)** | **0.60 (0.54-0.66)** | **0.78 (0.73-0.85)** |
| Petroleum products (n=9) | 839 | 1308 | **1.35 (1.13-1.62)** | 0.93 (0.74-1.17) | **0.56 (0.46-0.66)** |
| Complex organic fluids (n=7) | 743 | 1353 | **1.42 (1.17-1.73)** | **1.35 (1.08-1.69)** | 0.99 (0.84-1.17) |
| Gases (n=7) | 534 | 929 | **1.58 (1.28-1.94)** | **0.50 (0.25-0.99)** | **0.58 (0.47-0.70)** |
| Acids and bases (n=9) | 545 | 989 | 0.97 (0.80-1.18) | 1.20 (0.90-1.60) | 0.91 (0.74-1.11) |
| Organic dusts (n=31) | 1890 | 2838 | **1.25 (1.11-1.41)** | **0.85 (0.74-0.97)** | **0.88 (0.79-0.98)** |
| Inorganic dusts (n=24) | 3109 | 4558 | **1.21 (1.11-1.32)** | **0.62 (0.56-0.69)** | **1.13 (1.03-1.23)** |
| Radiation, electric and magnetic fields (n=2) | 784 | 841 | **2.78 (2.24-3.44)** | — | 0.95 (0.76-1.19) |
| General categories (n=5) | 1221 | 2053 | **1.29 (1.12-1.49)** | **0.36 (0.30-0.43)** | **0.77 (0.67-0.89)** |
| **By 2-digit CCDO (5)** |  |  |  |  |  |
| 41: Clerical and Related Occupations (n=10) | 535 | 716 | **1.36 (1.09-1.69)** | **0.34 (0.18-0.64)** | **0.42 (0.33-0.52)** |
| 51: Sales Occupations (n=10) | 688 | 1496 | **0.81 (0.68-0.96)** | **0.44 (0.29-0.67)** | **0.83 (0.70-0.99)** |
| 61: Service Occupations (n=14) | 2007 | 3461 | **1.16 (1.05-1.30)** | **0.61 (0.52-0.73)** | **0.87 (0.78-0.96)** |
| 71: Farming. Horticultural and Animal-Husbandry Occupations (n=5) | 1088 | 778 | **1.38 (1.15-1.65)** | **0.79 (0.65-0.96)** | **0.72 (0.61-0.86)** |
| 83: Machining and Related Occupations (n=5) | 1781 | 3370 | **1.34 (1.20-1.50)** | 1.00 (0.90-1.13) | 0.96 (0.86-1.07) |
| 85: Product Fabricating, Assembling and Repairing Occupations (n=9) | 3398 | 6401 | **1.20 (1.10-1.31)** | **1.22 (1.12-1.32)** | 0.96 (0.89-1.04) |
| 87: Construction Trades Occupations (n=12) | 5233 | 7566 | **1.18 (1.10-1.26)** | **0.74 (0.68-0.80)** | 0.95 (0.89-1.01) |
| 91: Transport Equipment Operating Occupations (n=10) | 2010 | 2665 | **1.93 (1.71-2.17)** | **0.29 (0.23-0.38)** | **0.78 (0.70-0.88)** |
| 93: Material-Handling and Related Occupations, n.e.c. (n=7) | 685 | 757 | 1.20 (0.99-1.46) | **0.44 (0.34-0.56)** | **1.59 (1.29-1.97)** |
| **By time period (n) (6)** |  |  |  |  |  |
| 1953-1993 (n=84) | 15647 | 26532 | **1.24 (1.19-1.29)** | **0.92 (0.88-0.96)** | **0.93 (0.89-0.96)** |
| 1934-1972 (n=40) (7) | 8775 | 9074 | **1.29 (1.22-1.37)** | **0.90 (0.84-0.96)** | 0.96 (0.91-1.01) |
| 1973-2012 (n=40) (7) | 6331 | 12824 | **1.14 (1.08-1.21)** | **0.80 (0.75-0.86)** | 1.02 (0.96-1.07) |

1. Number of unique exposures (job/agent pairs) in the Lung study data
2. Number of unique exposures (job/agent pairs) in the PROtEuS data
3. Cumulative odds ratio (95% confidence interval)
4. Chemical/Physical groups with at least 50 concordant exposed cells shown; n=number of agents within group
5. 2-digit CCDO groups with at least 50 concordant exposed cells shown; n=number of 7-digit occupations within 2-digit group
6. n represents the number of 7-digit occupations included in the time period
7. Data restricted to the same set of 7-digit occupations in the two time periods

Table S5. Agreement in exposure status among cells between Lung and PROtEuS exposure data

|  |  | **Percent concordant** | | **Percent discordant** | | **Concordant exposed cells** | | |
| --- | --- | --- | --- | --- | --- | --- | --- | --- |
|  | **Number of cells (1)** | **Exposed (%)** | **Unexposed (%)** | **Exposed Lung (%)**  **(2)** | **Exposed PROtEuS (%)**  **(3)** | **Number of cells (4)** | **Kendall correlation in probability**  **(5)** | **Median diff. in probability, PROtEuS-Lung (6)** |
| **Overall** | 18270 | 8.2 | 85.5 | 4.1 | 2.2 | 1502 | 0.55 | 1.3 |
| **Occupations with expert-annotated JEPs (n=75) (7)** | 15225 | 9.1 | 84.5 | 4.0 | 2.3 | 1393 | 0.55 | 1.5 |
| **By chemical group (8)** |  |  |  |  |  |  |  |  |
| Metals (n=21) | 1890 | 10.3 | 83.8 | 4.3 | 1.7 | 194 | 0.60 | 0.1 |
| Organic Solvents (n=17) | 1530 | 9.3 | 83.1 | 5.0 | 2.6 | 142 | 0.59 | 1.2 |
| Paints, varnishes and inks (n=8) | 720 | 8.1 | 84.7 | 5.3 | 1.9 | 58 | 0.58 | 2.2 |
| Combustion products and engine emissions (n=19) | 1710 | 17.1 | 74.1 | 5.3 | 3.5 | 293 | 0.54 | 0.2 |
| Petroleum products (n=11) | 990 | 8.2 | 84.1 | 3.9 | 3.7 | 81 | 0.34 | 4.9 |
| Complex organic fluids (n=8) | 720 | 9.7 | 82.8 | 4.2 | 3.3 | 70 | 0.38 | 11.5 |
| Gases (n=12) | 1080 | 5.0 | 89.2 | 4.1 | 1.8 | 54 | 0.48 | -1.2 |
| Acids and bases (n=15) | 1350 | 4.4 | 89.9 | 4.7 | 1.0 | 59 | 0.44 | 1.1 |
| Organic dusts (n=41) | 3690 | 4.7 | 90.3 | 3.4 | 1.6 | 174 | 0.60 | 0.0 |
| Inorganic dusts (n=25) | 2250 | 9.2 | 84.7 | 3.5 | 2.6 | 208 | 0.53 | 5.1 |
| Radiation, electric and magnetic fields (n=2) | 180 | 20.6 | 70.6 | 5.6 | 3.3 | 37 | 0.69 | 0.0 |
| General categories (n=5) | 450 | 21.3 | 63.3 | 8.2 | 7.1 | 96 | 0.55 | 2.4 |
| **By 2-digit CCDO major group (9)** |  |  |  |  |  |  |  |  |
| 41: Clerical and Related (n=10) | 2030 | 2.7 | 92.1 | 4.0 | 1.3 | 54 | 0.44 | -5.3 |
| 51: Sales (n=10) | 2030 | 4.3 | 90.6 | 2.1 | 3.0 | 88 | 0.43 | 10.5 |
| 61: Service (n=14) | 2842 | 5.5 | 89.7 | 3.3 | 1.5 | 157 | 0.54 | 2.0 |
| 71: Farming. Horticultural and Animal-Husbandry (n=5) | 1015 | 7.2 | 87.3 | 1.0 | 4.5 | 73 | 0.53 | 0.0 |
| 83: Machining and Related (n=5) | 1015 | 15.0 | 75.1 | 7.3 | 2.7 | 152 | 0.63 | -0.6 |
| 85: Product Fabricating, Assembling and Repairing (n=9) | 1827 | 15.3 | 76.0 | 6.3 | 2.4 | 279 | 0.57 | 0.9 |
| 87: Construction Trades (n=12) | 2436 | 17.0 | 74.7 | 6.1 | 2.2 | 414 | 0.54 | 3.7 |
| 91: Transport Equipment Operating (n=10) | 2030 | 5.0 | 91.1 | 2.4 | 1.6 | 101 | 0.62 | -2.2 |
| 93: Material-Handling and Related, n.e.c. (n=7) | 1421 | 6.0 | 84.2 | 6.8 | 3.0 | 85 | 0.53 | -1.3 |
| **By time period (10)** |  |  |  |  |  |  |  |  |
| 1953-1993 (n=84) | 17052 | 8.2 | 85.3 | 4.3 | 2.2 | 1400 | 0.56 | 1.4 |
| 1934-1972 (n=40) (e) | 6880 | 8.8 | 83.8 | 4.2 | 3.2 | 604 | 0.58 | 0.4 |
| 1973-2012 (n=40) (e) | 6880 | 9.2 | 82.1 | 5.8 | 3.0 | 631 | 0.58 | 1.9 |

1. Number of cells (occupation-agent combinations) with at least 10 jobs in both PROtEuS and Lung
2. Proportion of cells with probability ≥5% in Lung and <5% in PROtEuS
3. Proportion of cells with probability ≥5% in PROtEuS and <5% in Lung
4. Number of concordant exposed cells (probability ≥5% in Lung and ≥5% in PROtEuS)
5. Kendall correlation in the probability of exposure between concordant exposed cells
6. Median difference in probability (probability in PROtEuS minus probability in Lung) across concordant exposed cells
7. Restricted to cells with occupations with an expert-annotated JEP (75 7-digit CCDO)
8. Chemical/Physical groups with at least 50 concordant exposed cells shown; n=number of agents within group
9. 2-digit CCDO groups with at least 50 concordant exposed cells shown; n=number of 7-digit occupations within 2-digit group
10. n represents the number of 7-digit occupations included in the time period
11. Data restricted to the same set of 7-digit occupations in the two time periods
